# Targeting an ASCL1–WNT11–ERK feed-forward loop to overcome pyrotinib resistance in HER2-positive breast cancer

**DOI:** 10.1016/j.mtbio.2026.103679

**Published:** 2026-09-17

**Authors:** Fang Yin, Boxuan Zhou, Yingliang Li, Zide Liu, Chao Zhu, Yu Wang, Chuang Meng, Zhanglin Zhang, Alan Jiang, Wei Liu, Hongfei Liu, Xiaocheng Mao, Hong Tang, Tianmei Fu, Linwei Fan, Chengqi Gao, Kuai Yu, Qi Zeng, Aiping Le

**Affiliations:** a Department of Transfusion Medicine, Key Laboratory of Jiangxi Province for Transfusion Medicine, The First Affiliated Hospital, Jiangxi Medical College, Nanchang University, China; b Department of Breast Disease Center, The First Affiliated Hospital, The First Clinical Medical College, Jiangxi Medical College, Nanchang University, China; c Postdoctoral Innovation Practice Base, The First Affiliated Hospital, Jiangxi Medical College, Nanchang University, China; d Department of Gastroenterology, Digestive Disease Hospital, The First Affiliated Hospital of Nanchang University, China; e Medical Research Public Service Center, The First Affiliated Hospital, Jiangxi Medical College, Nanchang University, China; f Department of Neurology, The First Affiliated Hospital, Jiangxi Medical College, Nanchang University, China; g Department of Transfusion Medicine, The Second Affiliated Hospital, Jiangxi Medical College, Nanchang University, China

**Keywords:** Pyrotinib resistance, HER2-Positive breast cancer, ASCL1, siRNA co-delivery, ZIF-8 nanoparticles

## Abstract

Pyrotinib resistance remains a therapeutic obstacle in human epidermal growth factor receptor 2-positive (HER2^+^) breast cancer (BC), and tumor-selective strategies to restore drug response are lacking. Here, we identify ASCL1 as a mediator of pyrotinib resistance. ASCL1 was elevated in resistant cells and pretreatment tumors from pyrotinib-resistant patients. High ASCL1 expression was associated with shorter overall survival after adjustment for TNM stage. Mechanistically, ASCL1 promotes WNT11 transcription, thereby engaging non-canonical WNT11–ROR2 signaling to sustain ERK phosphorylation under pyrotinib treatment. Persistent ERK activity promotes CREB1 phosphorylation and p-CREB1 binding to the ASCL1 promoter and enhances its transcription, forming an ERK–CREB1–ASCL1–WNT11 feed-forward loop that reinforces resistance. To overcome this resistance program, we develop HApt-PEG-ZIF-8@siASCL1/Pyrotinib, a HER2 aptamer-guided ZIF-8 nanoplatform for tumor-selective co-delivery of ASCL1 siRNA and pyrotinib. This nanoplatform exhibits minimal hemolysis, enhanced uptake in HER2^+^ BC cells, prolonged circulation, and improved tumor delivery efficiency in vivo. Co-delivery suppresses ASCL1/WNT11 signaling and downstream p-ERK and p-CREB1, inhibits proliferation, migration, and invasion, induces apoptosis, and achieves robust antitumor efficacy in pyrotinib-resistant xenografts without detectable systemic toxicity. Together, this study reveals an ASCL1-driven resistance mechanism and establishes a HER2-targeted nanotherapeutic strategy to restore pyrotinib sensitivity, supporting ASCL1 as a therapeutic target in HER2^+^ BC.

## Highlights

• ASCL1 is elevated in pyrotinib-resistant HER2^+^ BC and linked to shorter survival.
• ASCL1 confers resistance and knockdown restores sensitivity in vitro and in vivo.
• ASCL1–WNT11–ROR2 sustains ERK; ERK–CREB1 reinforces ASCL1 transcription.
• HApt-PEG-ZIF-8 co-delivery restores pyrotinib response in resistant models.

## Graphical abstract

**Figure:**
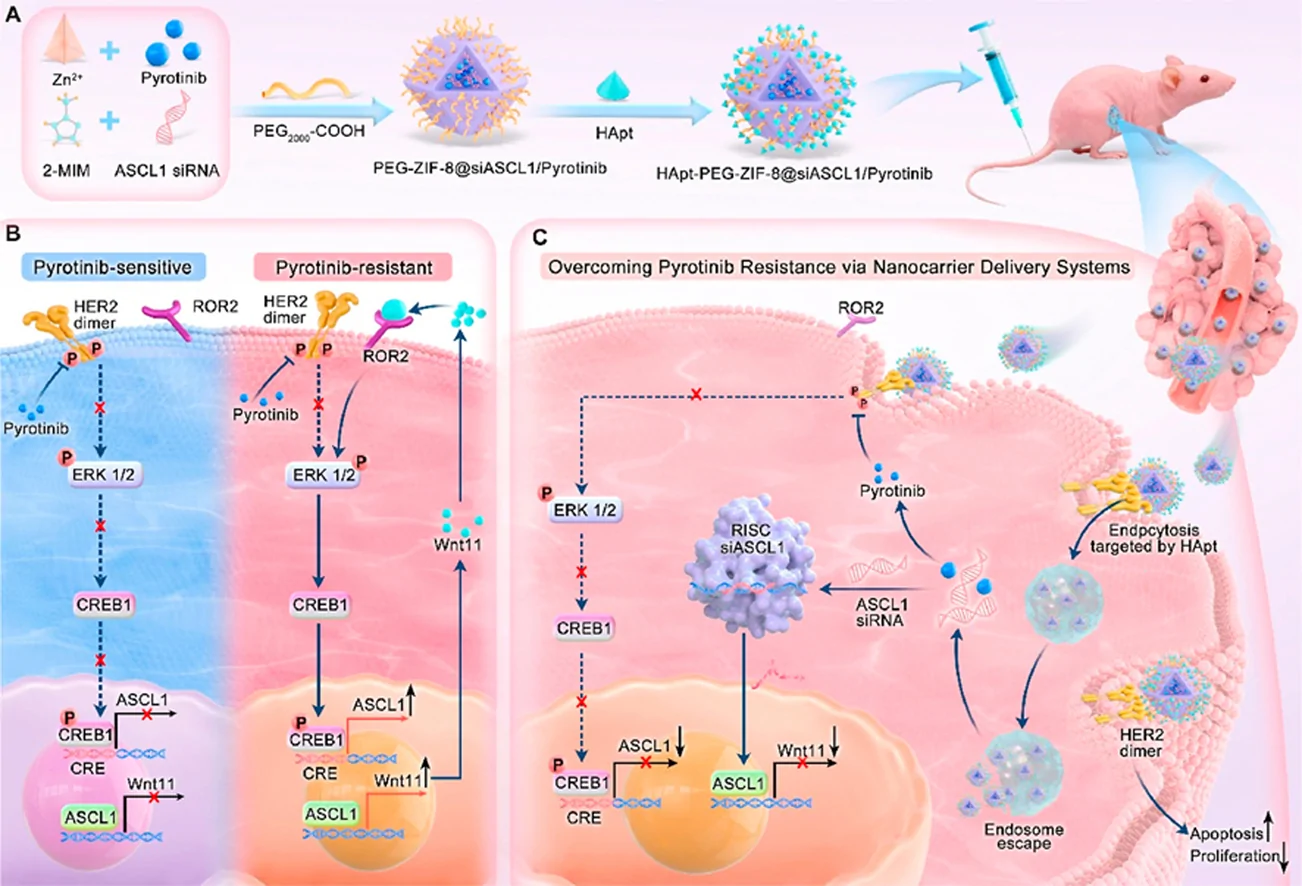

## 1 Introduction

HER2-positive (HER2^+^) breast cancer (BC) represents approximately 15–20% of breast cancer cases [1]. HER2 overexpression drives PI3K–AKT and RAS–RAF–MEK–ERK signaling, contributing to aggressive tumor behavior [2]. HER2-directed antibodies, antibody–drug conjugates, and tyrosine kinase inhibitors (TKIs) have markedly improved outcomes, but metastatic HER2^+^ BC remains incurable, with primary and acquired resistance limiting durable disease control [2,3]. Pyrotinib is an oral, irreversible pan-ErbB TKI targeting EGFR, HER2, and HER4 [4]. The phase II PICTURE study showed clinically meaningful activity of pyrotinib plus capecitabine in HER2^+^ advanced BC with primary trastuzumab resistance [5]. The final analysis of the phase III PHILA trial reported a sustained progression-free survival benefit and longer overall survival with first-line pyrotinib, trastuzumab, and docetaxel [6]. Despite these benefits, many patients with advanced disease ultimately experience progression, and the mechanisms limiting the durability of pyrotinib-based therapy remain incompletely understood.

Resistance to HER2-directed therapy involves activation of bypass signaling, shifts in downstream pathway dependence, and epigenetic, metabolic, or drug-tolerant cell-state adaptations [[7], [8], [9], [10], [11]]. Because most of these mechanisms were defined using HER2-directed agents other than pyrotinib, their relevance to acquired pyrotinib resistance remains uncertain [2,3]. Available pyrotinib-specific studies have preliminarily linked PIK3CA or TP53 mutations detected in baseline circulating tumor DNA to lower response and have implicated sustained PI3K–AKT signaling and PPARG-driven fatty acid metabolism in resistant models [4,12,13]. The transcriptional programs that maintain established pyrotinib resistance and the therapeutic vulnerabilities they may create remain poorly defined.

ASCL1 is a lineage-defining basic helix–loop–helix transcription factor and an oncogenic dependency in high-grade neuroendocrine cancers, where it regulates tumor-cell identity and survival [14,15]. In BC models, ASCL1 has also been linked to chemotherapy resistance, and its knockdown increases paclitaxel sensitivity in vitro and in xenografts [16,17]. Whether ASCL1 supports survival during HER2 inhibition or becomes a dependency in pyrotinib-resistant cells remains unknown. In small-cell lung cancer, ASCL1 promotes WNT11 expression through enhancer-associated regulation [18]. In BC, WNT11 interacts with receptor tyrosine kinase-like orphan receptor 2 (ROR2) and activates non-canonical signaling associated with tumor-cell invasion [19]. Whether ASCL1-dependent WNT11/ROR2 signaling contributes to acquired pyrotinib resistance remains unknown.

Because ASCL1 is difficult to inhibit selectively with conventional small molecules, siRNA-mediated knockdown offers a direct alternative but requires protection from degradation and clearance, efficient tumor-cell uptake, and cytosolic release [20,21]. Co-delivering pyrotinib and siASCL1 could couple continued HER2 blockade with ASCL1 knockdown in the same tumor cells [22]. Zeolitic imidazolate framework-8 (ZIF-8), a metal–organic framework, can load and protect nucleic acids and disassemble under acidic conditions to release its cargo [23], while ZIF-8-based hybrid systems have enabled small-molecule/siRNA co-delivery in drug-resistant cancer models [24,25]. Polyethylene glycol (PEG) surface modification can reduce nonspecific protein adsorption and macrophage interactions, while the HER2-binding HB5 aptamer (HApt) preferentially recognizes HER2^+^ BC cells [26,27].

Here, comparative transcriptomic profiling of pyrotinib-sensitive and -resistant cells identified ASCL1 as a functional mediator of acquired pyrotinib resistance. Mechanistically, ASCL1 induced WNT11–ROR2 signaling, which sustained ERK activity. ERK-dependent CREB1 activation, in turn, supported ASCL1 transcription, forming an ERK–CREB1–ASCL1–WNT11/ROR2 feed-forward loop. We therefore developed HApt-PEG-ZIF-8@siASCL1/Pyrotinib for HER2-directed co-delivery of siASCL1 and pyrotinib. The formulation sensitized resistant cells to pyrotinib and suppressed tumor growth in resistant xenografts.

## 2 Materials and methods

### 2.1 Tissue specimens

A total of 146 formalin-fixed, paraffin-embedded (FFPE) HER2^+^ BC specimens were obtained from the Department of Pathology, the First Affiliated Hospital of Nanchang University. All samples were from patients with histologically confirmed primary HER2^+^ BC who underwent surgical resection between March 2015 and March 2022. Clinicopathological data were abstracted from medical records, and detailed characteristics are provided in Table S1. OS was defined as the time from pathological diagnosis to death or was censored at the date of last follow-up. Within this cohort, 11 patients had complete records of pyrotinib-based combination therapy in the advanced salvage setting and serial radiological assessments. The IHC specimens from these patients were pretreatment primary tumor samples obtained before the initiation of pyrotinib-based therapy. Tumor response was assessed according to RECIST version 1.1 [28]. Patients achieving complete or partial response after at least two cycles of pyrotinib-based therapy were classified as pyrotinib-sensitive, whereas those with progressive disease during treatment were classified as pyrotinib-resistant. The study was approved by the Ethics Committee of the First Affiliated Hospital of Nanchang University, and written informed consent was obtained from all participants (Approval No. (2025)CDYFYYLK(01-035)).

### 2.2 Online dataset

The association between the expression of five candidate genes identified by RNA sequencing and overall survival (OS) was analyzed in the HER2^+^ breast cancer cohort of the KM-Plotter online database (https://kmplot.com/analysis/) [29]. For each gene, patients were divided into groups with high or low expression according to the auto-selected optimal cutoff in KM-Plotter, and Kaplan–Meier survival curves, hazard ratios (HRs) with 95% confidence intervals (CIs), and log-rank *P* values were obtained using the default settings.

### 2.3 Cell lines and cell culture

HEK-293T cells and the human BC cell lines SKBR3, MCF-7, and MDA-MB-231 were obtained from the Cell Bank of the Chinese Academy of Sciences (Shanghai, China). SKBR3-Res was a pyrotinib-resistant cell line established by continuous exposure of parental SKBR3 cells to increasing concentrations of pyrotinib for approximately 8 months. SKBR3-Res cells were cultured in drug-free medium for one week before experiments. All cell lines were cultured in DMEM (Gibco, USA) supplemented with 10% fetal bovine serum (Cellmax, China) and maintained at 37 °C in a humidified atmosphere with 5% CO2.

### 2.4 Chemicals

Pyrotinib dimaleate (SHR-1258) was obtained from Hengrui Medicine Co., Ltd. (China), dissolved in dimethyl sulfoxide (DMSO), and stored at −20 °C. The MEK inhibitor GSK1120212 was purchased from Selleck Chemicals (S2673, USA), and the MEK inhibitor U0126 was purchased from MedChemExpress (HY-12031A, USA). Actinomycin D was purchased from Yeasen Biotechnology (Shanghai) Co., Ltd. (53600ES08, China).

### 2.5 RNA extraction and quantitative real-time PCR (qRT-PCR)

Total RNA was extracted using TRIzol reagent (Servicebio, Wuhan, China) according to the manufacturer's instructions. For each sample, 1 μg of total RNA was reverse transcribed into cDNA using the TransScript® II All-in-One First-Strand cDNA Synthesis SuperMix for qPCR (TransGen Biotech, China). Transcript levels were quantified with ChamQ Universal SYBR qPCR Master Mix (Vazyme, China). Relative gene expression was calculated using the 2^−ΔΔCT^ method, with β-actin as the internal control. Primer sequences are listed in Table S4.

For mRNA stability analysis, SKBR3-Res cells were pretreated with DMSO, U0126 (10 μM), or GSK1120212 (5 μM) for 30 min, followed by the addition of actinomycin D (10 μg/mL). Cells were collected at 0, 1, 2, 4, and 6 h, with the corresponding treatments maintained throughout the chase. *ASCL1* mRNA levels were measured by qRT-PCR and normalized to the corresponding value at 0 h within each group.

### 2.6 siRNA, shRNA, plasmid construction, and cell transfection

Small interfering RNA (siRNA) and short hairpin RNA (shRNA) primers were synthesized by General Biosystems (Anhui) Co., Ltd. (Anhui, China), and their sequences are listed in Table S4. Human ASCL1 cDNA (RefSeq NM_004316.4) with a C-terminal HA tag was cloned into the lentiviral expression vector pLenti-CMV-GFP-BSD to generate pLenti-CMV-ASCL1-HA-GFP-BSD. Human WNT11 cDNA (RefSeq NM_004626.3) with a C-terminal HA tag was cloned into the same vector to generate pLenti-CMV-WNT11-HA-GFP-BSD. The lentiviral expression plasmids pLV3-CMV-CREB (human)-myc-Neo and pLV3-CMV-ROR2 (human)-3×Flag-Neo were purchased from Miaoling Biotechnology (Wuhan, China). All plasmid constructs were verified by Sanger sequencing before use. To generate stable cell lines with gene knockdown or overexpression, HEK293T cells were co-transfected with the target lentiviral plasmid together with pMD2.G and psPAX2 at a 4:1:3 ratio for lentiviral packaging. Six hours after transfection, the medium was replaced with fresh complete medium. Viral supernatants were collected 48 h later and passed through a 0.45-μm Steriflip filter. For transduction, the filtered viral supernatant supplemented with polybrene (10 μg/mL; Solarbio, Beijing, China) was used to infect breast cancer cells, followed by selection with puromycin (Solarbio, Beijing, China) or blasticidin S (Yeasen Biotechnology, Shanghai, China).

### 2.7 Cell viability assay

A CCK-8 cell viability assay was performed to identify the 50% inhibitory concentration (IC_50_). Cells were seeded in a 96-well plate at a density of 5000 cells/well and cultured overnight; cells were then treated with pyrotinib at 0, 1, 2, 4, 8, 16, 32, 64, 128, and 256 nM for 72 h. After 72 h of treatment, Cell Counting Kit-8 (CCK-8) reagent (GLPBIO, USA) was added at a concentration of 10%, and cells were incubated in a CO2 incubator for 2 h; OD_450_ was measured. The cell inhibitory rate was calculated as (OD_450_ negative control − OD_450_ treatment)/(OD_450_ negative control − OD_450_ blank control), and the 50% inhibitory concentration was determined as the IC_50_ of the drug.

### 2.8 Cell proliferation assay and colony formation assay

Cells were seeded in a 96-well plate at a density of 5 × 10^3^ cells per well and cultured overnight. Cells were then treated with DMSO or pyrotinib. Every 24 h, cells were incubated with CCK-8 for 2 h, and OD_450_ was measured. The experiment was performed three times, and cell proliferation curves were generated.

In addition, cell proliferation was analyzed by measuring DNA synthesis using the BeyoClick™ EdU Cell Proliferation Kit with AF555 (Beyotime, China). Briefly, cells were cultured in 96-well plates and treated with DMSO or pyrotinib for 72 h. Cells were then treated with 10 μM EdU for 4 h, followed by fixation, permeabilization, and staining with EdU working solution, and nuclei were stained with DAPI. EdU^+^ proliferative cells were observed under a fluorescence microscope.

For the colony formation assay, cells were cultured in a 6-well plate at a density of 1000 cells/well, and different treatments were added the next day. The culture medium containing the corresponding drugs was renewed every three days, and cells were cultured for three weeks. Finally, cells were fixed with 4% paraformaldehyde and stained with 0.1% Crystal Violet. Colonies (>50 cells/colony) were counted and compared with the control group. The experiment was performed in triplicate, and the data are representative of three separate experiments.

### 2.9 Transwell assay

For invasion assays, the upper chambers were precoated with Matrigel (Corning, Cat #354234) and allowed to solidify before seeding cells; migration assays were performed without Matrigel. Cells (1 × 10^5^) suspended in 200 μL serum-free medium containing different drug treatments were transferred into the upper chamber of 24-well transwell chambers (Cat#3422, Corning, NY, USA), and 600 μL medium containing 20% FBS was added to the lower chamber. The chamber was incubated at 37 °C for a specified period to facilitate cell migration or invasion through the membrane. After incubation for 48 h, cells that crossed the membrane were fixed with 4% PFA and stained with 0.1% Crystal violet. For each sample, five fields of view were obtained, and cell numbers were counted. The experiment was performed in triplicate, and the data are representative of three separate experiments.

### 2.10 Cell apoptosis assay

Apoptosis was assessed using either an Annexin V-APC/PI kit (BestBio, Cat#BB-41033) or an Annexin V-FITC/PI kit (Abbkine, Cat#KTA0006). Cells were collected after 72 h of treatment and washed twice with PBS. Cells were then resuspended in buffer, stained with APC or FITC for 10 min, and stained with PI for 5 min prior to flow cytometry analysis. All assays were performed in triplicate, and the data were obtained from three separate experiments.

### 2.11 Western blot

Protein samples were subjected to 8–12% SDS-PAGE according to protein size and transferred to nitrocellulose membranes (Millipore, USA). Membranes were then blocked with 5% milk for 2 h and incubated with primary antibodies overnight at 4 °C, followed by incubation with horseradish peroxidase-conjugated secondary antibodies for 2 h at room temperature. The UVP/ChemStudio Imaging System (Analytik Jena AG, Germany) was used to capture images. Primary antibody information is listed in Table S5.

### 2.12 Immunohistochemistry (IHC) and immunofluorescence (IF)

IHC was performed according to a standard protocol. FFPE tissue sections were deparaffinized, rehydrated, and subjected to antigen retrieval using sodium citrate buffer (pH 6.0) or EDTA buffer (pH 9.0) in a microwave. Subsequently, sections were blocked with 3% hydrogen peroxide and 5% bovine serum albumin (BSA), followed by incubation with primary antibodies overnight at 4 °C. The next day, sections were incubated with secondary antibodies for 1 h, stained with DAB solution, and counterstained with hematoxylin. Tissue sections were semi-quantitatively evaluated based on the proportion of positively stained tumor cells and staining intensity. The proportion of positive cells was scored as follows: 0 (0%), 1 (1–24%), 2 (25–49%), 3 (50–74%), and 4 (75–100%). Staining intensity was scored as follows: 0 (negative), 1 (weak), 2 (moderate), and 3 (strong). The proportion and intensity scores were multiplied to produce a total immune reactivity score of 0–12. We used the median value as the cut-off to define ASCL1-high and ASCL1-low in HER2^+^ breast cancer samples for ASCL1 expression analysis. Primary antibodies used for IHC are listed in Table S6.

For IF staining, tumor sections were similarly prepared and antigen retrieval was performed. Sections were blocked with 5% BSA for 30 min and incubated with WNT11 (Huabio, Cat#AC026) and ROR2 (Santa Cruz, Cat#sc-374174) antibodies overnight at 4 °C, followed by incubation with corresponding fluorescently labeled secondary antibodies for 1 h. Nuclei were stained with DAPI. Imaging was conducted using a ZEISS confocal laser scanning microscope (Germany). Co-localization was quantified using Pearson's coefficients in ImageJ (Coloc2 plugin) under identical threshold settings across samples.

### 2.13 Chromatin immunoprecipitation (ChIP)

Chromatin immunoprecipitation (ChIP) was performed using the Simple ChIP® Enzymatic Chromatin IP Kit (Magnetic Beads) (Cell Signaling Technology, #9003, USA) according to the manufacturer's protocol. Briefly, 4 × 10^6^ cells were cross-linked with 1% formaldehyde and quenched with glycine. Cross-linked chromatin was digested with the enzyme cocktail provided in the kit to generate predominantly mono- and oligonucleosome-sized fragments, and the digested chromatin was briefly sonicated to disrupt nuclear membranes and homogenize the lysate, yielding DNA fragments predominantly in the 200–1000 bp range. Chromatin was incubated overnight at 4 ^°^C with control IgG (Cell Signaling Technology, Cat#2729), anti-ASCL1 (Cell Signaling Technology, Cat#43666), or anti-phospho-CREB1 (Ser133) (Cell Signaling Technology, Cat#9198) antibodies. Protein G magnetic beads were added to each immunoprecipitation reaction and incubated for 2 h at 4 ^°^C with rotation. Immunocomplexes were eluted, and cross-links were reversed with proteinase K treatment followed by DNA purification according to the kit protocol. ChIP-enriched DNA was quantified by qPCR, and primer sequences are listed in Table S4.

### 2.14 Luciferase assay

Putative ASCL1-and CREB1-binding motifs in the WNT11 and ASCL1 promoters, respectively, were predicted using the JASPAR database [30], with a relative profile score threshold of 80%. The ASCL1 and CREB1 motifs were based on the JASPAR matrices MA1631.1 and MA0018.2, respectively. The WNT11 promoter fragment was PCR-amplified from human genomic DNA and cloned into the PGL3-Basic luciferase reporter vector. The wild-type and motif-mutant ASCL1 promoter reporter constructs, designated PGL3-ASCL1-WT and PGL3-ASCL1-Mut, respectively, were synthesized by Nanjing Jekesaisi Biotechnology Co., Ltd. (Nanjing, China). The ASCL1 promoter fragment corresponded to chr12:102,955,686–102,957,685 on the positive strand of the GRCh38/hg38 human genome assembly. In PGL3-ASCL1-Mut, the predicted CREB1-binding motif 5′-TGAGGCCA-3′ was replaced with 5′-TCTAAACA-3′. All constructs were verified by Sanger sequencing. HEK-293T cells or SKBR3-Res cells were co-transfected with the indicated reporter plasmids, the Renilla control plasmid pRL-TK, and the indicated expression plasmids using Lipofectamine 3000 (Thermo Fisher Scientific, USA) according to the manufacturer's instructions. Where indicated, SKBR3-Res cells were treated with U0126 (10 μM) or GSK1120212 (5 μM) for 24 h before luciferase measurement, with DMSO as the vehicle control. Luciferase activity was measured 48 h after transfection using the Dual Luciferase Reporter Gene Assay Kit (Yeasen Biotechnology, Shanghai, China) following the manufacturer's protocol. Firefly luciferase activity was normalized to Renilla luciferase activity, and normalized values were expressed relative to the PGL3-Basic empty vector control. Primer sequences are provided in Table S4.

### 2.15 Xenograft tumor in nude mice

Animal experiments received approval from the Animal Experimentation Ethics Committee of the First Affiliated Hospital of Nanchang University (Ethics Number: CDYFY-IACUC-202407QR230). Four-week-old female BALB/c nude mice were purchased from Hangzhou Ziyuan Laboratory Animal Technology Co., Ltd. Mice were injected subcutaneously into the flank with luciferase-expressing SKBR3-Sen cells stably overexpressing ASCL1 or the vector control (1 × 10^7^ cells per mouse; n = 12 per implantation group), suspended in Matrigel (Corning, Cat. No. 354234) (1:1). Once tumor volume reached approximately 100 mm^3^, mice were randomized into four groups (n = 6 per subgroup): Vector + DMSO, Vector + pyrotinib, OE-ASCL1 + DMSO, and OE-ASCL1 + pyrotinib. Mice were gavaged once daily with vehicle (DMSO) or pyrotinib (10 mg/kg) for 21 days. Tumor volume and body weight were measured every other day after injection, and tumor volume was calculated using the formula: V = (width^2^ × length)/2. Tumors were harvested at the end of treatment (day 28 after inoculation) and collected for immunohistochemical analysis.

For bioluminescence imaging, mice received D-luciferin (150 mg/kg) (Yeasen Biotechnology, Shanghai, China) and were imaged using an IVIS system (PerkinElmer) at 10–15 min post-injection. Total photon flux was quantified using Living Image software with identical ROI settings.

### 2.16 Synthesis of nanoparticles

ZIF-8 nanoparticles were synthesized via a precipitation method. Pyrotinib and siRNA were loaded into pre-synthesized ZIF-8 nanoparticles using an impregnation method, followed by PEGylation. To enable HER2 targeting, an anti-HER2 aptamer (HB5; 5′-AACCGCCCAAATCCCTAAGAGTCTGCACTTGTCATTTTGTATATGTATTTGGTTTTTGGCTCTCACAGACACACTACACACGCACA-3′) was synthesized by Sangon Biotech (Shanghai, China) and covalently conjugated to the PEG-modified nanoparticle surface via EDC/NHS chemistry.

### 2.17 Characterization of nanoparticles

The hydrodynamic diameter, polydispersity index (PDI), and zeta potential of the nanoparticles were measured using a Zetasizer Nano ZS90 (Malvern Instruments, UK). The morphological characteristics of the nanoparticles were analyzed by transmission electron microscopy (TEM). Following each loading or conjugation step, the nanoparticles were separated by centrifugation, and the resulting supernatant was collected for separate analysis. Ultraviolet–visible (UV–Vis) absorption spectra of the standard solutions and respective supernatants were recorded using a UV-1800 spectrophotometer (Shimadzu, Japan). Unencapsulated Cy5-labeled siASCL1, unencapsulated pyrotinib, and unconjugated HApt in the respective supernatants were quantified using calibration curves constructed from the corresponding standard solutions (5–80 μg/mL at 646.5 nm, 1–10 μg/mL at 260.5 nm, and 1–10 μg/mL at 261 nm, respectively). The encapsulation efficiency (EE) and loading capacity (LC) of siASCL1 and pyrotinib were calculated as follows: EE (%) = [(M_*input*_ − M_*free*_)/M_*input*_] × 100%; LC (%) = [(M_*input*_ − M_*free*_)/M_*NP*_] × 100%, where M_*input*_ represents the initially added mass of the respective cargo, M_*free*_ represents the mass of unencapsulated cargo remaining in the corresponding post-loading supernatant, and M_*NP*_ represents the mass of the dried nanoparticle formulation. The HApt conjugation efficiency was calculated as follows: conjugation efficiency (%) = [(M_*input*_ − M_*supernatant*_)/M_*input*_] × 100%. For this calculation, M_*input*_ represents the initially added mass of HApt, and M_*supernatant*_ represents the mass of unconjugated HApt remaining in the post-conjugation supernatant.

To verify HApt conjugation, a DNA marker, free HApt, PEG-ZIF-8@siASCL1/Pyrotinib, and HApt-PEG-ZIF-8@siASCL1/Pyrotinib were subjected to electrophoresis on a 2% agarose gel in TAE buffer at 100 V for 25 min. Nucleic acids were visualized using a nucleic acid stain (Servicebio, Wuhan, China), and images were acquired using the UVP/ChemStudio Imaging System (Analytik Jena AG, Germany).

### 2.18 In vitro release of pyrotinib and Cy5-siASCL1

HApt-PEG-ZIF-8@Cy5-siASCL1/Pyrotinib suspension (1 mL) was placed in a dialysis bag and immersed in 30 mL of PBS at pH 7.4, 6.5, or 5.5. The samples were incubated at 37 °C with shaking at 200 rpm. At predetermined time points over 48 h, 1 mL of the release medium was withdrawn and replaced with an equal volume of fresh PBS. The concentrations of released Cy5-siASCL1 and pyrotinib were determined using their respective calibration curves, based on fluorescence intensity measured with a microplate reader and absorbance at 260.5 nm measured with a UV–Vis spectrophotometer, respectively. Cumulative release was calculated with correction for serial sampling and buffer replacement.

### 2.19 Cellular uptake

MDA-MB-231, MCF-7, and SKBR3-Res cells were seeded in confocal dishes. After cell adhesion, cells were treated with bare ZIF-8, PEG-ZIF-8@Cy5-siASCL1/Pyrotinib, or HApt-PEG-ZIF-8@Cy5-siASCL1/Pyrotinib for 4 h. Cells were then incubated with LysoTracker Green (#C1047S, Beyotime) for 1 h and stained with Hoechst 33342 (1:1000) for 10 min. Fluorescence images were acquired using a confocal laser scanning microscope (ZEISS, Germany).

### 2.20 Hemolysis assay

Anticoagulated mouse blood was gently washed three times with PBS. After centrifugation, erythrocytes were resuspended in PBS (negative control), deionized water (positive control), or the indicated nanoparticle solutions. Suspensions were mixed thoroughly and incubated at 37 °C for 2 h. Samples were then centrifuged at 12,000 rpm for 10 min, and images of hemolysis were recorded. Absorbance of the supernatants was measured at 570 nm using a microplate reader.

### 2.21 Pharmacokinetics and biodistribution study

Healthy female BALB/c nude mice were used for the in vivo pharmacokinetic study (n = 3 per group). Mice received a single tail-vein injection of naked Cy5-siASCL1, PEG-ZIF-8@Cy5-siASCL1/Pyrotinib, or HApt-PEG-ZIF-8@Cy5-siASCL1/Pyrotinib, with an equivalent siASCL1 dose of 1 nmol per mouse. Blood samples were collected from the orbital vein into heparinized tubes at 0, 5, 30, 60, 120, 240, 360, 480, 600, and 720 min after injection. Fluorescence of Cy5-labeled siASCL1 in collected blood samples was measured using a microplate reader to assess pharmacokinetics.

For biodistribution analysis, female BALB/c nude mice bearing subcutaneous SKBR3-Res xenografts received the same three formulations by tail-vein injection at an equivalent siASCL1 dose of 1 nmol per mouse (n = 5 per group). At 24 h after injection, Cy5-siASCL1 fluorescence in tumors and major organs was quantified using a small-animal CT/live imaging all-in-one system (MILabs B.V.).

### 2.22 In vivo evaluation of antitumor efficacy and systemic toxicity

SKBR3-Res cells (1 × 10^7^ cells) were suspended in Matrigel (Corning, Cat. No. 354234) and injected subcutaneously into the flank of female BALB/c nude mice. When the tumor volume reached approximately 100 mm^3^, mice were randomized into six groups (n = 6 per group) and received tail-vein injections of PBS, ZIF-8, ZIF-8@siASCL1, ZIF-8@Pyrotinib, PEG-ZIF-8@siASCL1/Pyrotinib, or HApt-PEG-ZIF-8@siASCL1/Pyrotinib once every 3 days for a total of six administrations. At each administration, PEG-ZIF-8@siASCL1/Pyrotinib and HApt-PEG-ZIF-8@siASCL1/Pyrotinib were each dosed to deliver 2 nmol siASCL1 per mouse [23]. The co-delivered pyrotinib dose was determined from the measured pyrotinib-to-siASCL1 loading mass ratio of 1:2.99. The ZIF-8@siASCL1 and ZIF-8@Pyrotinib controls were dose-matched to the siASCL1 and pyrotinib doses in the dual-loaded formulations, respectively. At the end of the experiment, mice were euthanized, and major organs and tumors were collected and fixed in 4% paraformaldehyde for IHC and hematoxylin-eosin (H&E) staining. Blood samples were collected from mice, and serum biochemical parameters, including alanine aminotransferase (ALT), aspartate aminotransferase (AST), creatinine (CREA), blood urea nitrogen (BUN), and uric acid (UA), were measured using commercially available assay kits (Jiancheng BioTech, Nanjing, China) according to the manufacturer's instructions.

In a separate long-term biosafety study, healthy female BALB/c nude mice were assigned to the PBS, PEG-ZIF-8@siASCL1/Pyrotinib, or HApt-PEG-ZIF-8@siASCL1/Pyrotinib group (n = 6 per group). Mice received tail-vein injections once every 3 days for 70 days. The nanoparticle formulations were administered at an siASCL1-equivalent dose of 2 nmol per mouse, with the corresponding pyrotinib dose determined by the measured loading ratio. At the 70-day endpoint, blood samples were collected for serum ALT, AST, BUN, and CREA measurements, and the heart, liver, spleen, lung, and kidney were collected for H&E staining.

### 2.23 RNA sequencing and results analysis

RNA-seq was performed using three biological replicates per group (SKBR3-Sen cells and SKBR3-Res cells). Differentially expressed genes (DEGs) were identified using the criteria of |log_2_ fold change| > 1 and adjusted *P* value < 0.05. Genes with log_2_ fold change > 1 were considered upregulated, and those with log_2_ fold change < −1 were considered downregulated.

### 2.24 Statistical analysis

Statistical analyses were performed using R and GraphPad Prism. Data are presented as mean ± standard deviation (SD) from three independent experiments. Two-group comparisons were performed using unpaired *t* tests or Mann–Whitney *U* tests, as appropriate. Multiple-group comparisons were performed using one- or two-way ANOVA followed by Tukey's multiple-comparisons test. Overall survival was analyzed using Kaplan–Meier curves, log-rank tests, and univariate and multivariate Cox proportional hazards regression. Based on clinical relevance, the univariate results, and the number of outcome events (24 deaths), the multivariate Cox model included ASCL1 expression and TNM stage. Time-dependent ROC curves were generated using the survivalROC package. Categorical clinicopathologic variables were compared using χ^2^ or Fisher's exact tests. *ASCL1* mRNA decay curves were fitted using a one-phase decay model and compared by an extra sum-of-squares *F* test. A *P* value below 0.05 was considered statistically significant.

## 3 Results

### 3.1 ASCL1 is associated with pyrotinib resistance and poor prognosis in HER2^+^ BC

To investigate the mechanisms underlying pyrotinib resistance in HER2^+^ BC, a pyrotinib-resistant SKBR3 cell line (SKBR3-Res) was established from parental SKBR3 (SKBR3-Sen) cells through stepwise drug exposure. Compared with SKBR3-Sen cells, SKBR3-Res cells exhibited enlarged cell morphology and a more disorganized arrangement (Fig. S1A). Sensitivity to pyrotinib was significantly reduced in SKBR3-Res cells, as evidenced by a right-shifted dose–response curve and an increase in the IC_50_ value from 4.643 nM in SKBR3-Sen cells to 150.7 nM in SKBR3-Res cells (Fig. S1B), confirming the successful induction of resistance.

**Fig. 1:**
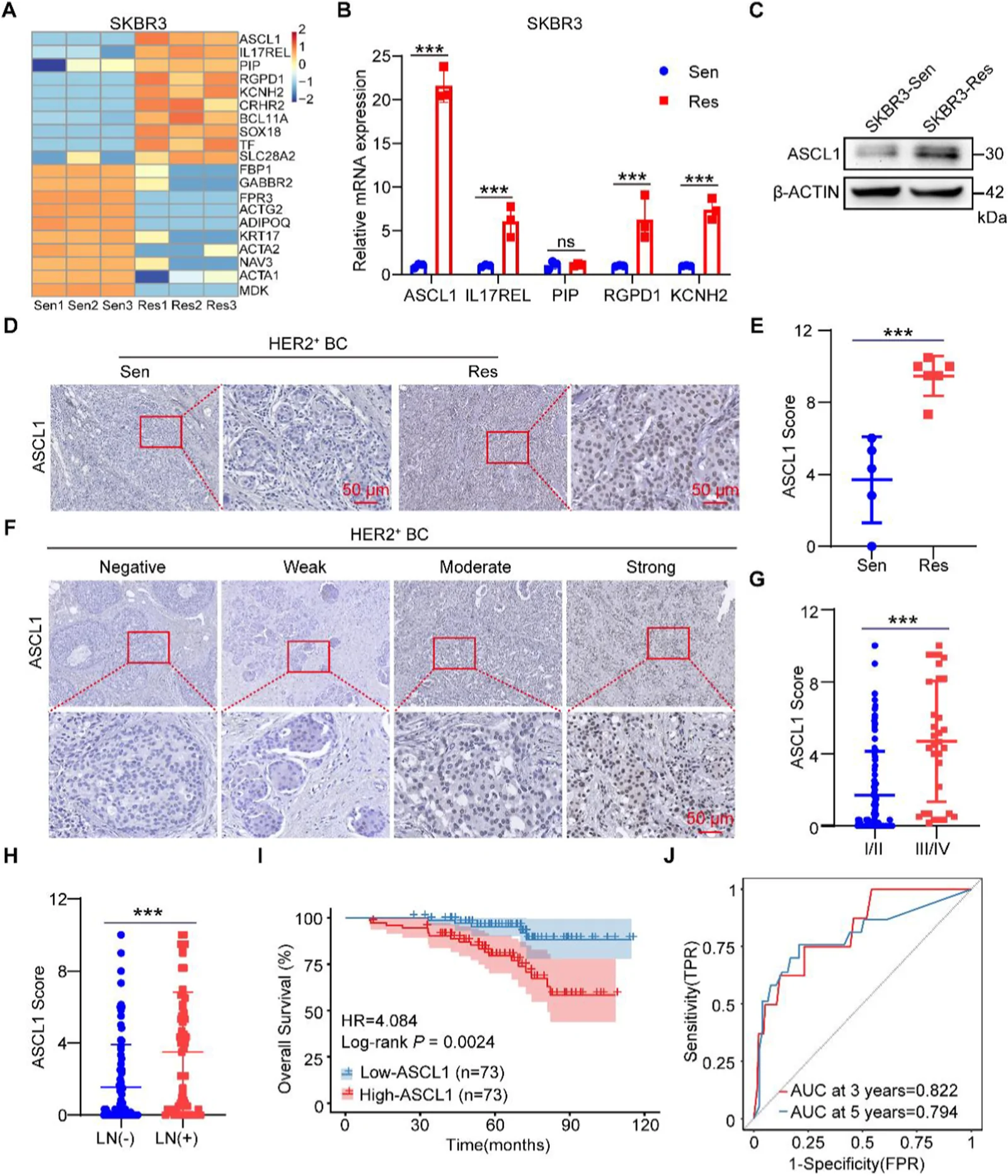
**ASCL1 is associated with pyrotinib resistance and poor prognosis in HER2^+^ BC. (A)** Heatmap of differentially expressed genes identified by RNA sequencing in pyrotinib-sensitive (SKBR3-Sen) and pyrotinib-resistant (SKBR3-Res) cells (Sen1–3 and Res1–3 denote biological replicates). **(B)** RT–qPCR validation of *ASCL1*, *IL1*7REL, *PIP*, *RGPD1*, and *KCNH2* mRNA levels in SKBR3-Sen and SKBR3-Res cells. **(C)** Immunoblot analysis of indicated proteins in SKBR3-Sen and SKBR3-Res cells. **(D)** Representative IHC images of ASCL1 staining in pretreatment primary tumor specimens from patients classified as pyrotinib-sensitive (Sen) or pyrotinib-resistant (Res) according to subsequent treatment response. Scale bar, 50 μm. **(E)** Semi-quantitative IHC scores of ASCL1 in pretreatment primary tumor specimens from pyrotinib-sensitive (n = 5) and pyrotinib-resistant (n = 6) patients. **(F)** Representative IHC images showing negative, weak, moderate, and strong ASCL1 staining in a cohort of HER2^+^ BC tissues (n = 146). Scale bar, 50 μm. **(G,H)** ASCL1 IHC scores according to TNM stage (I/II vs III/IV) (G) and lymph node status (LN^−^ vs LN^+^) (H) in the HER2^+^ BC cohort (n = 146). **(I)** Kaplan–Meier overall survival analysis stratified by ASCL1 expression (Low-ASCL1 vs High-ASCL1; n = 73 per group). **(J)** Time-dependent ROC analysis evaluating the prognostic performance of ASCL1 IHC scores for 3- and 5-year overall survival. Data are presented as mean ± SD (B) or individual values (E,G,H). Statistical analysis was performed as described in the Methods. ns, not significant. ***P < 0.001.

RNA sequencing was subsequently performed to identify genes associated with pyrotinib resistance. A set of differentially expressed genes was identified between SKBR3-Sen and SKBR3-Res cells, with ASCL1 showing the most prominent upregulation in resistant cells, alongside several other candidates, including IL17REL, PIP, RGPD1, and KCNH2 (Fig. 1A). RT–qPCR confirmed elevated mRNA levels of *ASCL1*, *IL1*7REL, *RGPD1*, and *KCNH2* in SKBR3-Res cells relative to SKBR3-Sen cells, whereas *PIP* expression did not differ significantly between the two cell lines (Fig. 1B). To assess the clinical relevance of these differentially expressed genes, we evaluated the association between their expression and overall survival in an independent HER2^+^ BC cohort using the KM-Plotter online database. Among the identified genes, only high ASCL1 expression was consistently associated with significantly shorter overall survival. By contrast, high expression of IL17REL, PIP, or KCNH2 was associated with improved overall survival, whereas RGPD1 expression showed no significant association with survival outcomes (Fig. S1C). Consistent with the RNA-seq and RT–qPCR data, immunoblot analysis confirmed that ASCL1 protein levels were higher in SKBR3-Res cells than in SKBR3-Sen cells (Fig. 1C). Collectively, these findings highlight ASCL1 as a promising candidate for further investigation in the context of pyrotinib resistance in HER2^+^ BC.

To assess whether ASCL1 upregulation is also observed in patient tumors, ASCL1 expression was evaluated by immunohistochemistry (IHC) in pretreatment primary tumor specimens from 11 patients with documented responses to pyrotinib-based therapy. ASCL1 staining was weak or nearly absent in specimens from pyrotinib-sensitive patients but was markedly stronger in those from pyrotinib-resistant patients (Fig. 1D). In this exploratory subset, semi-quantitative IHC scores were significantly higher in the resistant group than in the sensitive group (Fig. 1E). In the overall cohort of 146 HER2^+^ BC cases, ASCL1 expression was assessed by IHC (Fig. 1F). Based on the median IHC staining score, tumors were classified into ASCL1-low and ASCL1-high groups. The clinicopathological characteristics of the cohort are provided in Table S1. ASCL1 IHC scores were significantly higher in patients with TNM stage III–IV than in those with stage I–II and in patients with positive rather than negative lymph node status (Fig. 1G and H). Consistent with these score-based comparisons, categorical analyses of ASCL1-low and ASCL1-high groups are presented in Table S1. Kaplan–Meier analysis revealed that patients with high ASCL1 expression had significantly poorer overall survival than those with low expression (Fig. 1I). After adjustment for TNM stage, high ASCL1 expression remained associated with shorter overall survival (HR = 3.380, 95% CI 1.248–9.155, *P* = 0.017; Table S2). Time-dependent receiver operating characteristic (ROC) analysis further evaluated the prognostic performance of ASCL1 IHC scores for 3- and 5-year overall survival (Fig. 1J). Together, the 11-patient analysis provides exploratory evidence of an association between pretreatment ASCL1 expression and subsequent response to pyrotinib-based therapy, whereas the 146-patient cohort supports a TNM-adjusted association between high ASCL1 expression and shorter overall survival in HER2^+^ BC.

### 3.2 ASCL1 reduces pyrotinib sensitivity in HER2^+^ BC cells

**Fig. 2:**
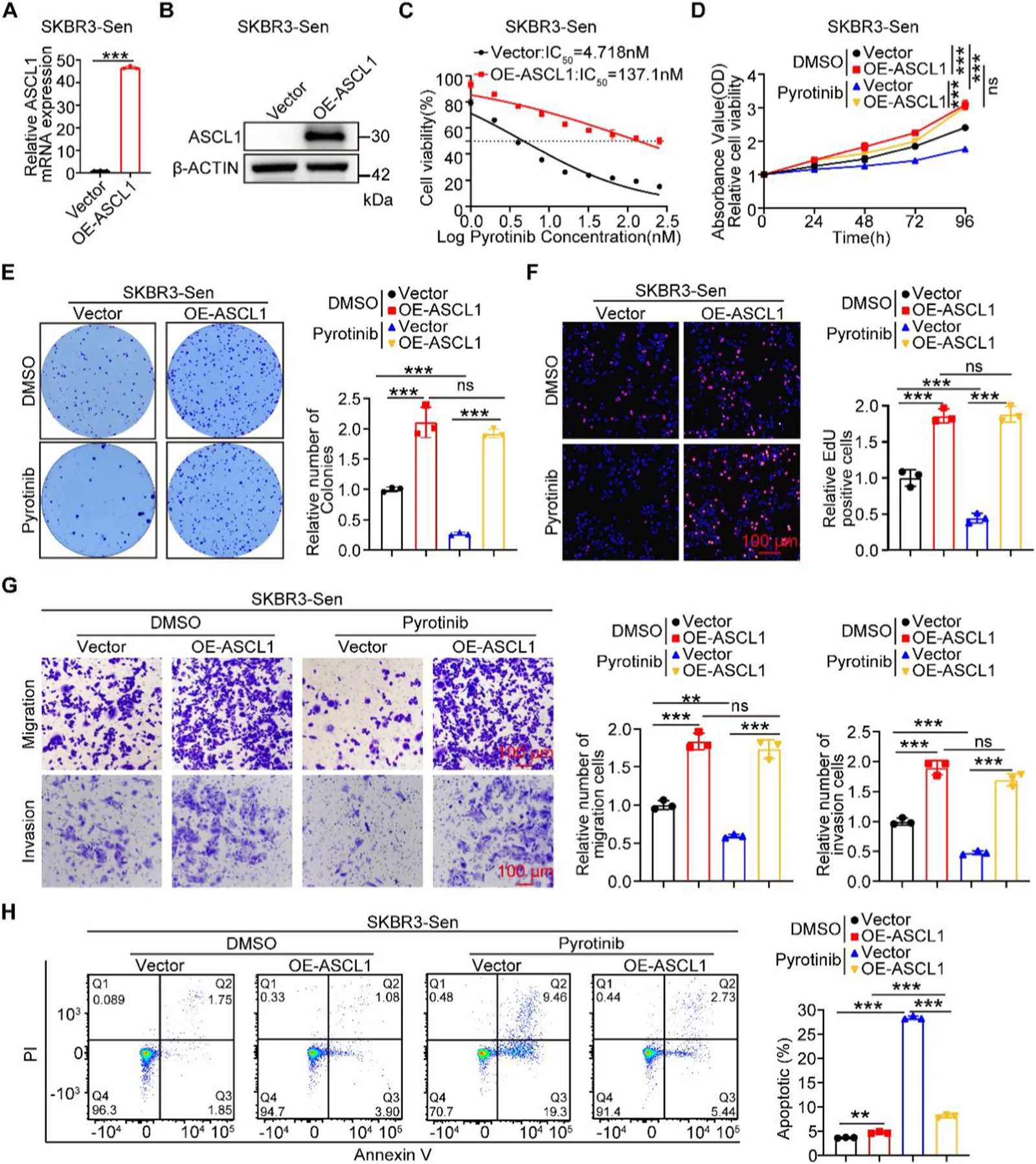
**ASCL1 reduces pyrotinib sensitivity in HER2^+^ BC cells. (A)** RT–qPCR analysis of *ASCL1* mRNA expression in SKBR3-Sen cells transduced with control vector or ASCL1-overexpressing lentivirus. **(B)** Immunoblot analysis of indicated proteins in SKBR3-Sen control and ASCL1-overexpressing cells. **(C)** Dose–response curves of SKBR3-Sen control and ASCL1-overexpressing cells treated with pyrotinib; IC_50_ values are indicated. **(D)** Cell viability of SKBR3-Sen vector and ASCL1-overexpressing cells treated with DMSO or pyrotinib, measured at the indicated time points. **(E)** Representative colony formation images and quantification in SKBR3-Sen control and ASCL1-overexpressing cells under DMSO or pyrotinib treatment. **(F)** EdU incorporation assay showing representative fluorescence images and quantification of EdU-positive cells in SKBR3-Sen vector and ASCL1-overexpressing cells treated with DMSO or pyrotinib. Scale bar, 100 μm. **(G)** Transwell migration and invasion assays showing representative images and quantification of SKBR3-Sen control and ASCL1-overexpressing cells treated with DMSO or pyrotinib. Scale bar, 100 μm. **(H)** Flow cytometric analysis of apoptosis by Annexin V/PI staining in SKBR3-Sen control and ASCL1-overexpressing cells treated with DMSO or pyrotinib, with quantification of apoptotic cells. Data are presented as mean ± SD. Statistical analysis was performed as described in the Methods. Brackets indicate the groups compared. ns, not significant. **P < 0.01, ***P < 0.001.

To investigate the role of ASCL1 in pyrotinib sensitivity, ASCL1 expression was modulated in HER2^+^ BC cell lines via lentiviral transduction. Immunoblotting and RT–qPCR confirmed successful ASCL1 overexpression in SKBR3-Sen cells and efficient knockdown in SKBR3-Res cells (Fig. 2A and B; Fig. S2A and B). In SKBR3-Sen cells, dose–response analysis revealed a pronounced rightward shift in the pyrotinib sensitivity curve, with the IC_50_ increasing from 4.718 nM in control group to 137.1 nM in ASCL1-overexpressing group (Fig. 2C). Under DMSO treatment, ASCL1 overexpression significantly enhanced cell viability compared with vector controls. In the presence of pyrotinib, ASCL1-overexpressing cells exhibited significantly higher viability than vector controls, suggesting that ASCL1 upregulation markedly attenuated the growth-inhibitory effects of pyrotinib (Fig. 2D). Consistently, colony formation and EdU incorporation assays showed that ASCL1 overexpression promoted colony formation and increased DNA synthesis compared with controls under both DMSO- and pyrotinib-treated conditions, with particularly pronounced effects under pyrotinib exposure (Fig. 2E and F). Transwell migration and invasion assays further demonstrated that ASCL1-overexpressing cells exhibited significantly higher migratory and invasive potential than vector controls under pyrotinib treatment (Fig. 2G). Conversely, pyrotinib-induced apoptosis was markedly diminished in ASCL1-overexpressing cells compared with vector controls, as evidenced by Annexin V/propidium iodide staining (Fig. 2H).

Opposite effects were observed when ASCL1 was silenced in SKBR3-Res cells. ASCL1 knockdown restored drug sensitivity, as reflected by a marked reduction in the IC_50_ for pyrotinib in SKBR3-Res cells, which decreased from 148.6 nM in sh-NC cells to 27.18 and 39.04 nM in the two ASCL1-silenced clones, respectively (Fig. S2C). Consistent with restored drug sensitivity, ASCL1 knockdown reduced basal cell viability and further enhanced the growth-suppressive effect of pyrotinib compared with sh-NC control cells (Fig. S2D). Colony formation and EdU assays demonstrated that ASCL1 knockdown significantly impaired clonogenic survival and DNA synthesis under both DMSO and pyrotinib treatments, with a more pronounced reduction under pyrotinib exposure (Fig. S2E and F). Migration and invasion were also substantially impaired following ASCL1 knockdown, especially under pyrotinib treatment (Fig. S2G), while apoptosis was concomitantly increased (Fig. S2H).

Collectively, these findings suggest that ASCL1 enhances proliferative, migratory, and invasive capacities while suppressing apoptosis in HER2^+^ BC cells, thereby contributing to reduced sensitivity to pyrotinib.

### 3.3 ASCL1 sustains pyrotinib resistance in HER2^+^ BC in vivo

**Fig. 3:**
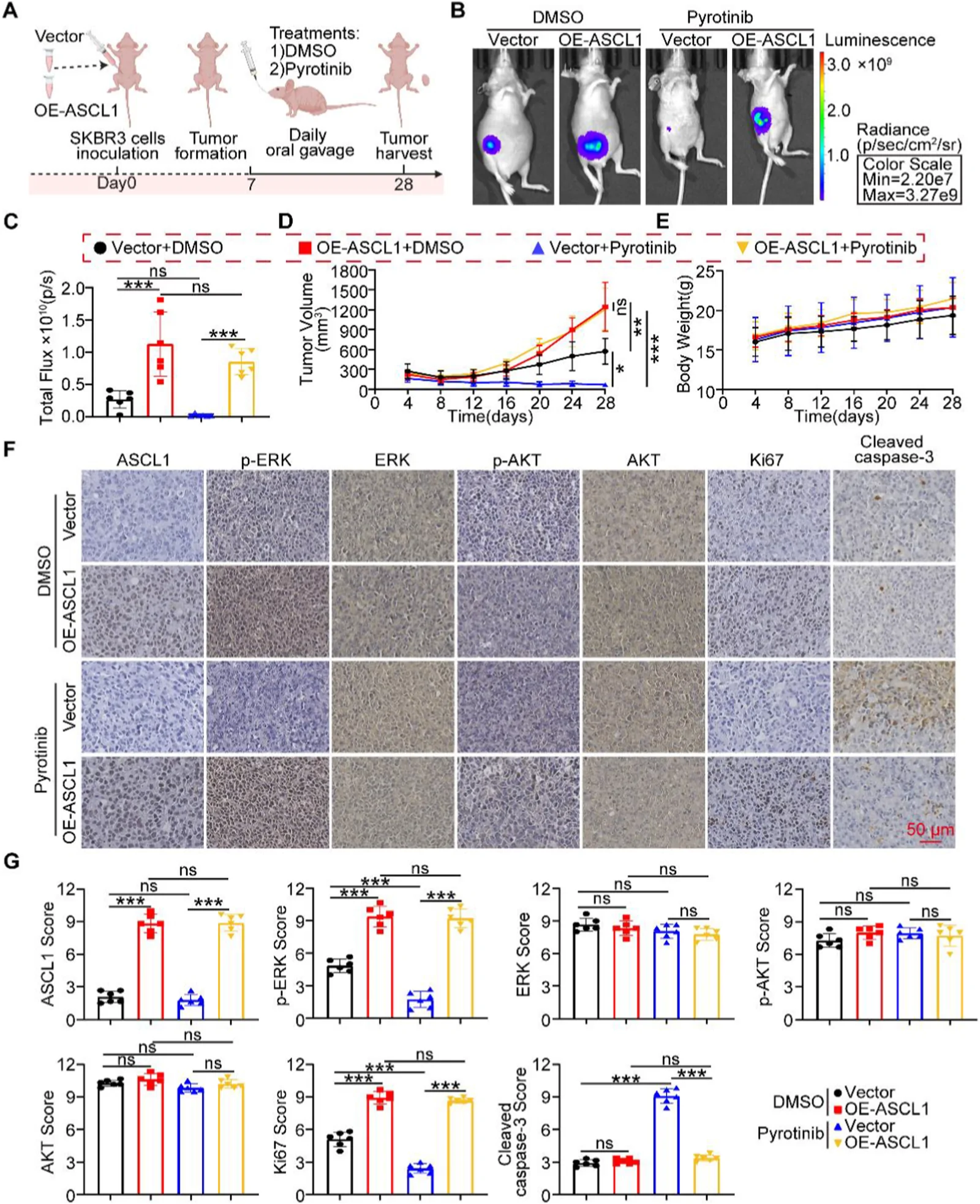
**ASCL1 sustains pyrotinib resistance in HER2^+^ BC in vivo. (A)** Schematic illustration of the xenograft study design (created with BioRender.com). Luciferase-labeled SKBR3 cells stably expressing empty vector or ASCL1 were inoculated into nude mice. When tumors reached approximately 100 mm^3^, mice were treated daily by oral gavage with DMSO or pyrotinib (10 mg/kg), and tumors were harvested on day 28. **(B)** Representative bioluminescence images of mice bearing vector or ASCL1-overexpressing xenografts under DMSO or pyrotinib treatment. **(C)** Quantification of bioluminescence signal shown as total photon flux (×10^10^ photons/s) in each group. **(D)** Tumor growth curves showing tumor volume changes over time in the four treatment groups. **(E)** Body weight curves of mice during treatment. **(F)** Representative IHC staining of indicated proteins in excised xenograft tumors from each group. Scale bar, 50 μm. **(G)** Semi-quantitative IHC scoring of indicated proteins in xenograft tumors. Data are presented as mean ± SD; each symbol represents one mouse (n = 6 mice per group). Statistical analysis was performed as described in the Methods. ns, not significant. Brackets indicate the groups compared. *P < 0.05, **P < 0.01, ***P < 0.001.

To further validate the in vitro findings, a xenograft model was established using SKBR3 cells stably transduced with ASCL1 or an empty vector. Luciferase-labeled cells were inoculated into nude mice. When tumor volumes reached approximately 100 mm^3^, mice were treated daily by oral gavage with DMSO or pyrotinib, resulting in four experimental groups: Vector + DMSO, Vector + pyrotinib, OE-ASCL1 + DMSO, and OE-ASCL1 + pyrotinib (Fig. 3A). Bioluminescence imaging showed that ASCL1-overexpressing tumors produced significantly stronger signals than vector-control tumors under DMSO treatment. Pyrotinib notably reduced the luminescent signal in control group, whereas ASCL1-overexpressing tumors maintained high photon flux in the presence of pyrotinib (Fig. 3B). Quantitative analysis confirmed that total flux in the pyrotinib-treated OE-ASCL1 group remained substantially higher than in pyrotinib-treated control group, approaching levels observed in DMSO-treated OE-ASCL1 xenografts (Fig. 3C).

Tumor volume measurements were consistent with the bioluminescence data. In the control group, pyrotinib treatment significantly slowed tumor growth compared with DMSO, leading to smaller final tumor volumes. In contrast, ASCL1-overexpressing tumors grew more rapidly than vector controls under DMSO treatment and continued to grow despite pyrotinib treatment, ultimately achieving the largest volumes among all experimental groups at the end of treatment (Fig. 3D). Body weights remained stable and comparable across all treatment groups throughout the study (Fig. 3E). These findings indicate that ASCL1 overexpression diminishes the in vivo antitumor effect of pyrotinib.

Given that TKI resistance is frequently associated with sustained MAPK and/or PI3K–AKT signaling, we performed IHC analysis of excised xenografts to assess pathway activity and tumor phenotypes. IHC staining confirmed robust nuclear accumulation of ASCL1 in tumors derived from OE-ASCL1 group, with minimal staining in vector-control tumors. Levels of phosphorylated ERK (p-ERK) were significantly higher in ASCL1-overexpressing xenografts compared with vector controls, regardless of treatment, whereas total ERK, phosphorylated AKT (p-AKT), and total AKT levels were comparable across groups. In vector tumors, pyrotinib reduced p-ERK staining, whereas p-ERK levels remained high in ASCL1-overexpressing tumors despite pyrotinib exposure, suggesting sustained ERK activation upon ASCL1 upregulation. Additionally, Ki-67 staining was more pronounced and cleaved caspase-3 staining was weaker in ASCL1-overexpressing tumors than in control group under pyrotinib treatment, consistent with increased cellular proliferation and reduced apoptosis (Fig. 3F and G).

Taken together, these in vivo data corroborate the cell-based experiments and support the conclusion that enforced ASCL1 expression is sufficient to attenuate the antitumor efficacy of pyrotinib in this xenograft model, in association with sustained ERK activation and a pro-proliferative, anti-apoptotic phenotype.

### 3.4 ASCL1 promotes pyrotinib resistance through WNT11–ROR2–ERK signaling

**Fig. 4:**
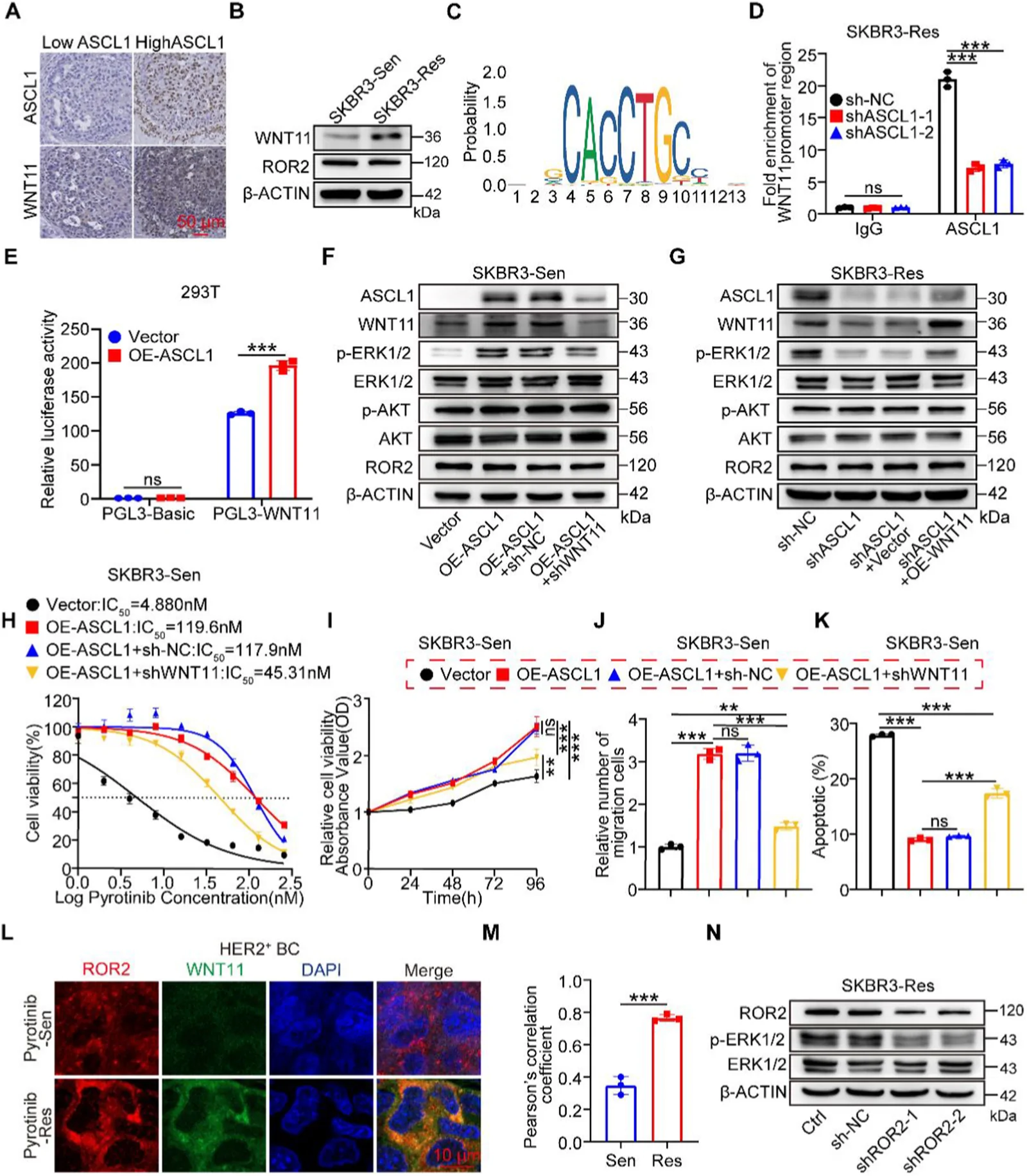
**ASCL1 promotes pyrotinib resistance through WNT11–ROR2–ERK signaling. (A)** Representative IHC staining of ASCL1 and WNT11 in clinical HER2^+^ BC tissues (n = 36). Scale bar, 50 μm. **(B)** Immunoblot analysis of indicated proteins in SKBR3-Sen and SKBR3-Res cells. **(C)** Predicted ASCL1-binding motif within the WNT11 promoter identified by the JASPAR database. **(D)** ChIP–qPCR analysis of ASCL1 occupancy at the WNT11 promoter in SKBR3-Res cells transduced with sh-NC or shASCL1. IgG served as a negative control. **(E)** Luciferase reporter assay in 293T cells transfected with the WNT11 promoter reporter (PGL3-WNT11) or empty reporter (PGL3-Basic) together with vector or ASCL1. **(F)** Immunoblot analysis of indicated proteins in SKBR3-Sen cells under pyrotinib treatment for 48 h. **(G)** Immunoblot analysis of indicated proteins in SKBR3-Res cells under pyrotinib treatment for 48 h. **(H)** Dose–response curves to pyrotinib in SKBR3-Sen cells with vector control, ASCL1 overexpression, and ASCL1 overexpression with WNT11 knockdown; IC_50_ values were calculated. **(I)** Cell viability of SKBR3-Sen cells with the indicated manipulations under pyrotinib treatment. **(J)** Quantification of transwell migration in SKBR3-Sen cells with the indicated manipulations under pyrotinib treatment. **(K)** Quantification of apoptosis (Annexin V/PI staining) in SKBR3-Sen cells with the indicated manipulations under pyrotinib treatment. **(L)** Representative immunofluorescence images showing ROR2 (red), WNT11 (green), and nuclei (DAPI, blue) in pretreatment primary tumor specimens from patients in the pyrotinib-sensitive and pyrotinib-resistant groups. Scale bar, 10 μm. **(M)** Quantification of WNT11–ROR2 co-localization from (L) (n = 3 independent specimens per group). **(N)** Immunoblot analysis of indicated proteins in SKBR3-Res cells following ROR2 knockdown. Data are presented as mean ± SD. Statistical analysis was performed as described in the Methods. Brackets indicate the groups compared. ns, not significant; **P < 0.01, ***P < 0.001.

Previous studies have identified WNT11 as a transcriptional target of ASCL1, with ASCL1-induced WNT11 playing a key role in neuroendocrine differentiation and cell proliferation in small-cell lung cancer [18]. Building on this, we hypothesized that ASCL1 promotes pyrotinib resistance in HER2^+^ BC by regulating WNT11 expression. IHC analysis revealed a positive correlation between ASCL1 and WNT11 expression in clinical HER2^+^ BC specimens (Fig. 4A; Fig. S3A). Similarly, WNT11 protein levels were significantly higher in SKBR3-Res cells than in SKBR3-Sen cells (Fig. 4B). A putative ASCL1-binding motif within the WNT11 promoter was identified using the JASPAR database (Fig. 4C). ChIP–qPCR analysis revealed that the WNT11 promoter region was significantly enriched in ASCL1 immunoprecipitates from SKBR3-Res cells, with this enrichment markedly reduced upon ASCL1 knockdown (Fig. 4D). Furthermore, luciferase assays showed that ASCL1 overexpression enhanced the activity of the PGL3-WNT11 promoter construct in 293T cells (Fig. 4E). These results collectively indicate that ASCL1 transcriptionally activates WNT11 in HER2^+^ BC cells.

To investigate the role of WNT11 in ASCL1-mediated pyrotinib resistance, SKBR3-Sen cells stably overexpressing ASCL1 were transduced with a WNT11-targeting shRNA (Fig. S3B), while SKBR3-Res cells with stable ASCL1 knockdown were engineered to overexpress WNT11. Immunoblot analysis showed that ASCL1 overexpression increased WNT11 and p-ERK levels in SKBR3-Sen cells. WNT11 knockdown partially attenuated the ASCL1-induced increase in p-ERK, with no significant change in p-AKT levels (Fig. 4F). In contrast, ASCL1 knockdown in SKBR3-Res cells decreased WNT11 and p-ERK, while WNT11 overexpression in ASCL1-knockdown SKBR3-Res cells partially restored p-ERK levels without affecting p-AKT expression (Fig. 4G). These findings suggest that ASCL1 enhances ERK activation primarily through WNT11, without substantial changes in AKT signaling. We further examined the effects of WNT11 on pyrotinib-related cellular phenotypes. In SKBR3-Sen cells, WNT11 knockdown partially reversed the ASCL1-driven increases in proliferation and migration, restored pyrotinib-induced apoptosis, and reduced the pyrotinib IC_50_ (Fig. 4H–K). Conversely, in SKBR3-Res cells, WNT11 overexpression partially counteracted the effects of ASCL1 knockdown on proliferation and migration, attenuated the increase in apoptosis, and increased the pyrotinib IC_50_ (Fig. S3C–F). Overall, these data suggest that ASCL1 contributes to pyrotinib resistance in HER2^+^ BC by sustaining ERK signaling via WNT11.

As a secreted WNT ligand, WNT11 can activate distinct intracellular signaling pathways depending on the receptor and co-receptor context of recipient cells [31]. In BC, WNT11 has been shown to physically interact with the cysteine-rich domain (CRD) of ROR2 [19], and ROR2-dependent non-canonical WNT signaling has been associated with ERK activation in cancer [32]. Based on these findings, we hypothesized that ROR2 may serve as a receptor for WNT11 in SKBR3-Res cells. To explore whether WNT11–ROR2 signaling contributes to sustained ERK phosphorylation and pyrotinib resistance, we performed immunofluorescence analysis in HER2^+^ BC specimens. Compared with pretreatment primary tumor specimens from the pyrotinib-sensitive group, the pyrotinib-resistant group showed increased membrane co-localization of WNT11 and ROR2 (Fig. 4L and M). To determine whether the WNT11–ROR2 interaction contributes to sustained ERK phosphorylation in the resistant state, ROR2 expression was modulated in SKBR3-Res cells by lentiviral transduction, and RT–qPCR confirmed efficient knockdown of ROR2 (Fig. S3G). Immunoblotting showed that ROR2 knockdown decreased p-ERK levels (Fig. 4N). In dose–response assays, ROR2 knockdown shifted the pyrotinib sensitivity curve leftward, resulting in reduced IC_50_ values compared with controls (Fig. S3H). Taken together, these findings suggest that the WNT11–ROR2 interaction plays an important role in maintaining sustained ERK phosphorylation, thereby contributing to reduced pyrotinib sensitivity in the resistant state.

### 3.5 ERK–CREB1 signaling sustains ASCL1 transcription and pyrotinib resistance

ASCL1 was persistently upregulated in pyrotinib-resistant cells and more highly expressed in pretreatment tumors from the resistant group (Fig. 1C–E). Because ASCL1-induced WNT11 sustained ERK phosphorylation (Fig. 4F and G), we next examined whether ERK activity feeds back to maintain ASCL1 transcription. CREB1 has been previously implicated in the regulation of ASCL1 transcription [33], and ERK signaling is known to enhance CREB1 transactivation via Ser133 phosphorylation [34]. Based on these findings, we hypothesized that CREB1 could act as a transcriptional intermediary linking ERK activity to the regulation of ASCL1 expression. Thus, we investigated whether ERK signaling sustains ASCL1 transcription through CREB1.

**Fig. 5:**
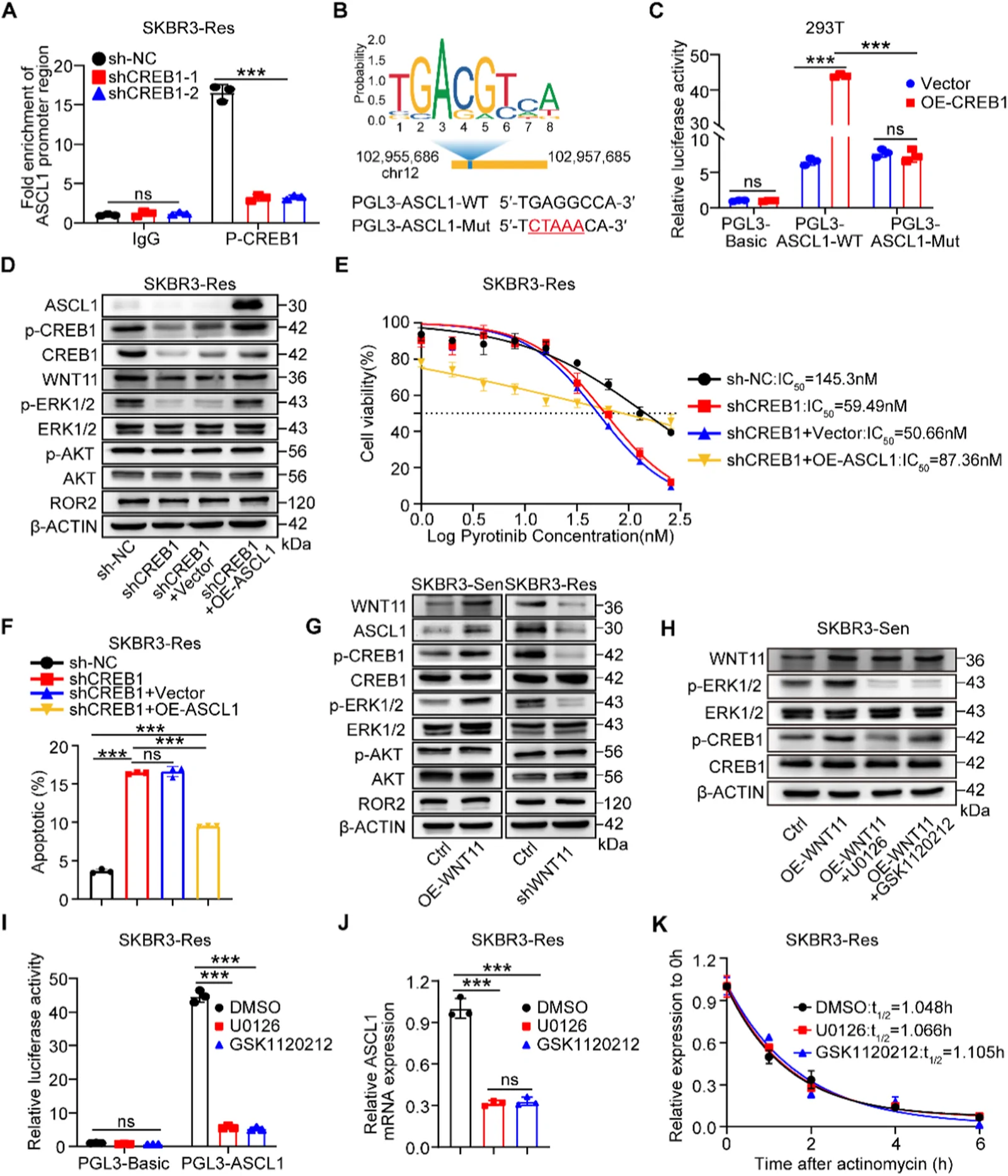
**ERK–CREB1 signaling sustains ASCL1 transcription and pyrotinib resistance. (A)** ChIP–qPCR analysis of phosphorylated CREB1 (p-CREB1) enrichment at the ASCL1 promoter in SKBR3-Res cells transduced with sh-NC, shCREB1-1, or shCREB1-2. IgG served as a negative control. **(B)** JASPAR-predicted CREB1-binding motif and schematic representation of the wild-type (WT) and motif-mutant (Mut) ASCL1 promoter reporters. Substituted nucleotides are highlighted in red. **(C)** Luciferase reporter assay in HEK-293T cells transfected with PGL3-Basic, PGL3-ASCL1-WT, or PGL3-ASCL1-Mut together with Vector or OE-CREB1. PGL3-Basic served as the promoterless control. **(D–F)** SKBR3-Res cells transduced with sh-NC or shCREB1, with or without ASCL1 re-expression, were analyzed as follows. **(D)** Immunoblot analysis of the indicated proteins after 48 h of pyrotinib treatment. **(E)** Pyrotinib dose–response curves; IC_50_ values are indicated. **(F)** Annexin V/PI analysis of apoptosis under pyrotinib treatment. **(G)** Immunoblot analysis of the indicated proteins in SKBR3-Sen cells with WNT11 overexpression or SKBR3-Res cells with WNT11 knockdown after 48 h of pyrotinib treatment. **(H)** Immunoblot analysis of the indicated proteins in control and WNT11-overexpressing SKBR3-Sen cells, with the latter treated with U0126 (10 μM) or GSK1120212 (5 μM) for 24 h. **(I, J)** SKBR3-Res cells were treated with DMSO, U0126 (10 μM), or GSK1120212 (5 μM) for 24 h. **(I)** ASCL1 promoter luciferase activity. **(J)** RT–qPCR analysis of *ASCL1* mRNA expression. **(K)** Actinomycin D (10 μg/mL) chase analysis of *ASCL1* mRNA decay in SKBR3-Res cells treated with DMSO, U0126, or GSK1120212. Relative *ASCL1* mRNA abundance was normalized to 0 h within each group. Data are presented as mean ± SD. Statistical analyses were performed as described in the Methods. Brackets indicate the groups compared. ns, not significant; ***P < 0.001.

We first examined whether ASCL1 transcription is regulated by CREB1 in pyrotinib-resistant cells. To this end, SKBR3-Res cells were transduced with shRNAs targeting CREB1, and RT–qPCR confirmed efficient CREB1 knockdown (Fig. S4A). ChIP–qPCR demonstrated significant enrichment of phosphorylated CREB1 (p-CREB1) at the ASCL1 promoter in sh-NC cells, which was markedly reduced following CREB1 knockdown (Fig. 5A). We next mutated the JASPAR-predicted CREB1-binding motif in the ASCL1 promoter from 5′-TGAGGCCA-3′ to 5′-TCTAAACA-3′ (Fig. 5B). CREB1 overexpression enhanced the activity of the wild-type ASCL1 promoter but did not significantly increase the activity of the motif-mutant reporter; the promoterless PGL3-Basic vector also remained at background activity (Fig. 5C). Immunoblot analysis revealed that CREB1 knockdown reduced p-CREB1 and led to decreased levels of ASCL1, WNT11, and p-ERK. Re-expression of ASCL1 in CREB1-knockdown cells restored WNT11 expression and p-ERK levels, while CREB1 protein levels remained suppressed (Fig. 5D). In line with these findings, ASCL1 overexpression in SKBR3-Sen cells resulted in a marked increase in *ASCL1* and *WNT1*1 mRNA levels, with minimal effects on CREB1 and ROR2 transcript levels (Fig. S4B). These data position CREB1 upstream of ASCL1 and link CREB1-dependent ASCL1 transcription to the activation of the WNT11/ERK signaling axis.

Functionally, CREB1 knockdown sensitized SKBR3-Res cells to pyrotinib, and ASCL1 re-expression partially rescued this phenotype. In dose–response assays, CREB1 knockdown lowered the pyrotinib IC_50_ value, whereas ASCL1 re-expression partially restored the IC_50_ value (Fig. 5E). In CCK-8 assays, CREB1 knockdown reduced cell viability under pyrotinib treatment, while ASCL1 re-expression partially restored cell viability (Fig. S4C). Transwell assays showed fewer migrated cells upon CREB1 knockdown, with partial rescue by ASCL1 overexpression (Fig. S4D). Annexin V/PI staining revealed increased apoptosis in CREB1-knockdown cells, which was partially reversed by ASCL1 re-expression (Fig. 5F). Collectively, these results support a functional role of the CREB1–ASCL1 axis in mediating pyrotinib resistance.

Given that ASCL1 directly activates WNT11, we next investigated whether CREB1 also regulates the WNT11 promoter. In contrast to the ASCL1 promoter, ChIP–qPCR analysis revealed no significant enrichment of p-CREB1 at the WNT11 promoter region compared with the IgG control, and this signal remained unchanged following CREB1 knockdown (Fig. S4E). Notably, CREB1 overexpression enhanced the activity of a WNT11 promoter reporter in 293T cells, and this enhancement was markedly reduced upon ASCL1 silencing (Fig. S4F). These findings suggest that CREB1 regulates WNT11 transcription primarily through ASCL1, with no detectable p-CREB1 enrichment at the examined WNT11 promoter region.

Finally, we explored whether WNT11 sustains the CREB1–ASCL1 axis by reinforcing ERK activity. In SKBR3-Sen cells, WNT11 overexpression increased ASCL1 protein levels and elevated both p-CREB1 and p-ERK levels. Conversely, WNT11 knockdown in SKBR3-Res cells reduced p-CREB1, ASCL1, and p-ERK levels (Fig. 5G). Together, these observations suggest that WNT11 may help maintain CREB1 activation and ASCL1 expression through ERK signaling. To test whether CREB1 Ser133 phosphorylation depends on ERK activity in this context, we treated WNT11-overexpressing SKBR3-Sen cells with two MEK1/2 inhibitors, U0126 and GSK1120212. Both inhibitors suppressed ERK phosphorylation and concomitantly reduced p-CREB1 levels (Fig. 5H). We then explored whether ERK activity supports ASCL1 transcriptional output. Consistently, U0126 and GSK1120212 markedly decreased ASCL1 promoter-driven luciferase activity in SKBR3-Res cells, while the PGL3-Basic control remained at background levels (Fig. 5I). Both inhibitors also significantly reduced endogenous *ASCL1* mRNA expression (Fig. 5J). To determine whether the reduction in *ASCL1* mRNA resulted from altered mRNA stability, *ASCL1* mRNA decay was evaluated using an actinomycin D chase assay. No significant differences in *ASCL1* mRNA decay were detected among the DMSO control, U0126, and GSK1120212 groups (*P* = 0.8202; Fig. 5K). These results indicate that MEK–ERK inhibition decreases ASCL1 expression primarily by suppressing its transcription rather than by accelerating *ASCL1* mRNA degradation. Taken together, our findings support a model in which WNT11 reinforces ERK activity, ERK-dependent CREB1 activation sustains ASCL1 transcription, which in turn drives WNT11 expression to maintain ERK phosphorylation and pyrotinib resistance.

### 3.6 Construction and characterization of HApt-PEG-ZIF-8@siASCL1/pyrotinib

**Fig. 6:**
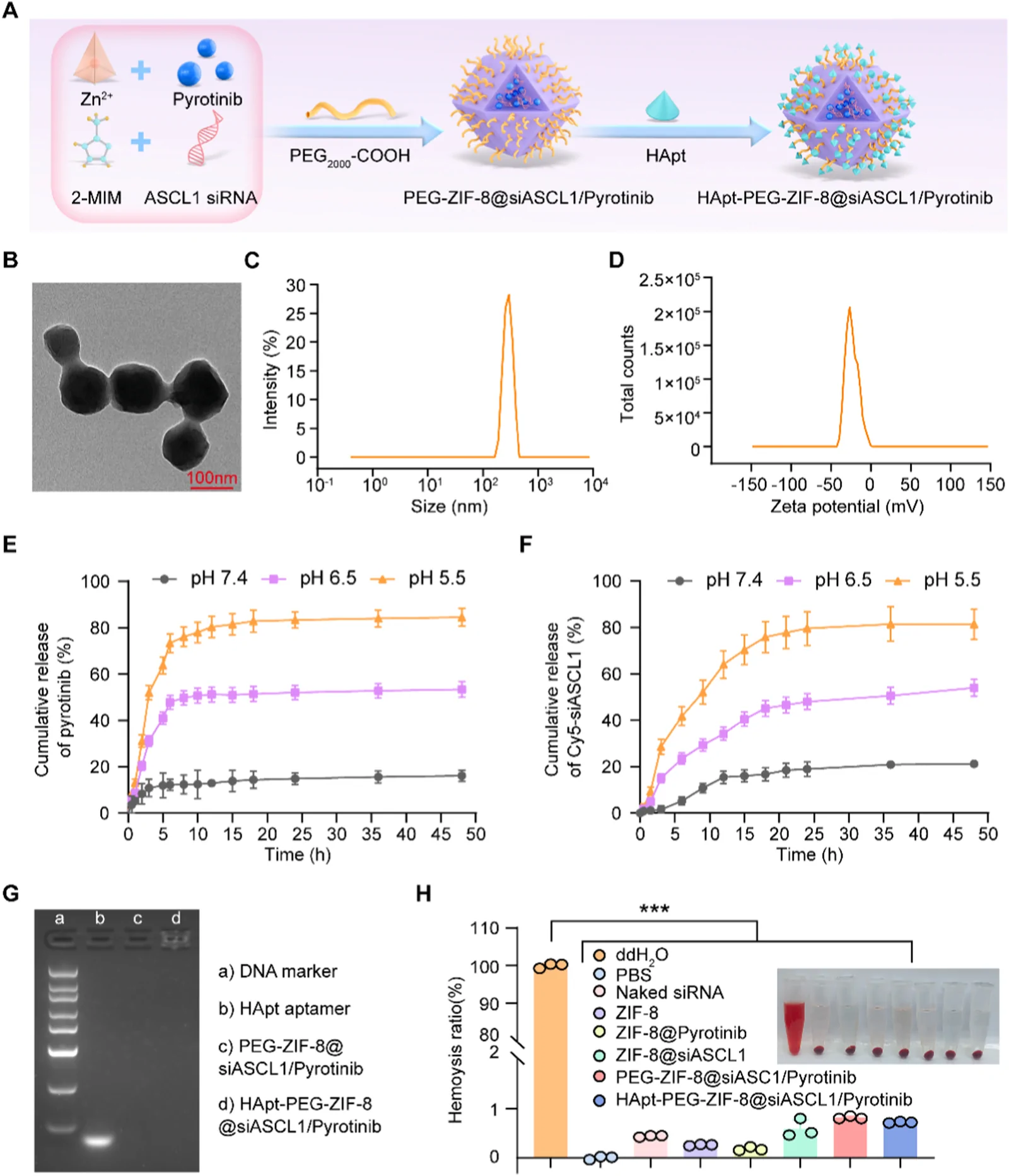
**Construction and characterization of HApt-PEG-ZIF-8@siASCL1/Pyrotinib. (A)** Schematic illustration of the preparation of HER2-targeted HApt-PEG-ZIF-8@siASCL1/Pyrotinib nanoparticles. **(B)** Transmission electron microscopy (TEM) image showing the morphology of PEG-ZIF-8@siASCL1/Pyrotinib nanoparticles. Scale bar, 100 nm. **(C)** Representative hydrodynamic size distribution of HApt-PEG-ZIF-8@siASCL1/Pyrotinib. **(D)** Representative zeta-potential distribution of HApt-PEG-ZIF-8@siASCL1/Pyrotinib. **(E, F)** Cumulative release profiles of pyrotinib (E) and Cy5-siASCL1 (F) from HApt-PEG-ZIF-8@siASCL1/Pyrotinib at pH 7.4, 6.5, and 5.5 over 48 h. **(G)** Agarose gel electrophoresis of the DNA marker (lane a), free HApt (lane b), PEG-ZIF-8@siASCL1/Pyrotinib (lane c), and HApt-PEG-ZIF-8@siASCL1/Pyrotinib (lane d). **(H)** Hemolysis assay evaluating the hemocompatibility of the indicated formulations; PBS and deionized water were used as negative and positive controls, respectively; all nanoparticle formulations were compared with the deionized-water positive control. Data are presented as mean ± SD. Statistical analysis was performed as described in the Methods. Brackets indicate the groups compared. ***P < 0.001.

To translate our mechanistic findings into a potential therapeutic strategy, we prepared a ZIF-8-based carrier for co-delivery of ASCL1 siRNA (siASCL1) and pyrotinib. As schematically illustrated, ZIF-8 nanoparticles were synthesized from zinc nitrate and 2-methylimidazole (2-MIM), loaded with pyrotinib followed by siASCL1, and then modified with PEG_2000_-COOH to generate PEG-ZIF-8@siASCL1/Pyrotinib. Subsequently, a HER2-targeting DNA aptamer (HApt) was conjugated to the PEGylated nanoparticles, yielding HApt-PEG-ZIF-8@siASCL1/Pyrotinib (Fig. 6A).

Transmission electron microscopy showed that PEG-ZIF-8@siASCL1/Pyrotinib nanoparticles exhibited a polyhedral morphology (Fig. 6B). The mean hydrodynamic diameter increased from 116.9 ± 10.6 nm for ZIF-8 to 344.9 ± 17.0 nm for HApt-PEG-ZIF-8@siASCL1/Pyrotinib, while the mean PDI values of the five formulations ranged from 0.044 to 0.280. ZIF-8 and ZIF-8@Pyrotinib were positively charged, whereas the siASCL1-containing formulations were negatively charged; the final HApt-functionalized formulation had a zeta potential of −23.53 ± 0.57 mV (Fig. 6C and D; Table S3). Based on UV–Vis quantification of unencapsulated pyrotinib and Cy5-labeled siASCL1 in the corresponding post-loading supernatants, the EE values of pyrotinib and siASCL1 were calculated to be 78.52% and 74.77%, respectively (Fig. S5A–D). The corresponding LC values were 1.62% and 4.84%, respectively, yielding a loaded pyrotinib-to-siASCL1 mass ratio of approximately 1:2.99. We next evaluated the release of pyrotinib and siASCL1 from HApt-PEG-ZIF-8@siASCL1/Pyrotinib at pH 7.4, 6.5, and 5.5, representing physiological extracellular pH, the extracellular pH of the tumor microenvironment, and endolysosomal pH, respectively. At 48 h, cumulative pyrotinib release increased from approximately 16% at pH 7.4 to 53% at pH 6.5 and further to 85% at pH 5.5 (Fig. 6E). Cumulative siASCL1 release likewise increased from approximately 21% at pH 7.4 to 54% at pH 6.5 and further to 81% at pH 5.5 (Fig. 6F). Thus, release of both payloads was limited at physiological pH, increased at the extracellular pH of the tumor microenvironment, and increased further at endolysosomal pH. Agarose gel electrophoresis showed a distinct band for free HApt. In contrast, HApt-PEG-ZIF-8@siASCL1/Pyrotinib was retained in the loading well, whereas no corresponding signal was detected for PEG-ZIF-8@siASCL1/Pyrotinib (Fig. 6G), supporting successful conjugation of HApt to the PEGylated nanoparticles. Based on UV–Vis quantification of unconjugated HApt remaining in the post-conjugation supernatant, the HApt conjugation efficiency was calculated to be 87.77% (Fig. S5E and F). The hemocompatibility of the ZIF-8 formulations was assessed using a red blood cell hemolysis assay. In contrast to deionized water, which induced almost complete hemolysis, all nanoparticle formulations exhibited hemolysis ratios below 1% (Fig. 6H), indicating minimal hemolytic activity under the tested conditions.

Collectively, these results demonstrated that HApt-PEG-ZIF-8@siASCL1/Pyrotinib nanoparticles were successfully fabricated, released both payloads in a pH-responsive manner, and exhibited low hemolytic activity in vitro.

### 3.7 Cellular uptake and in vitro activity of HApt-PEG-ZIF-8@siASCL1/pyrotinib

**Fig. 7:**
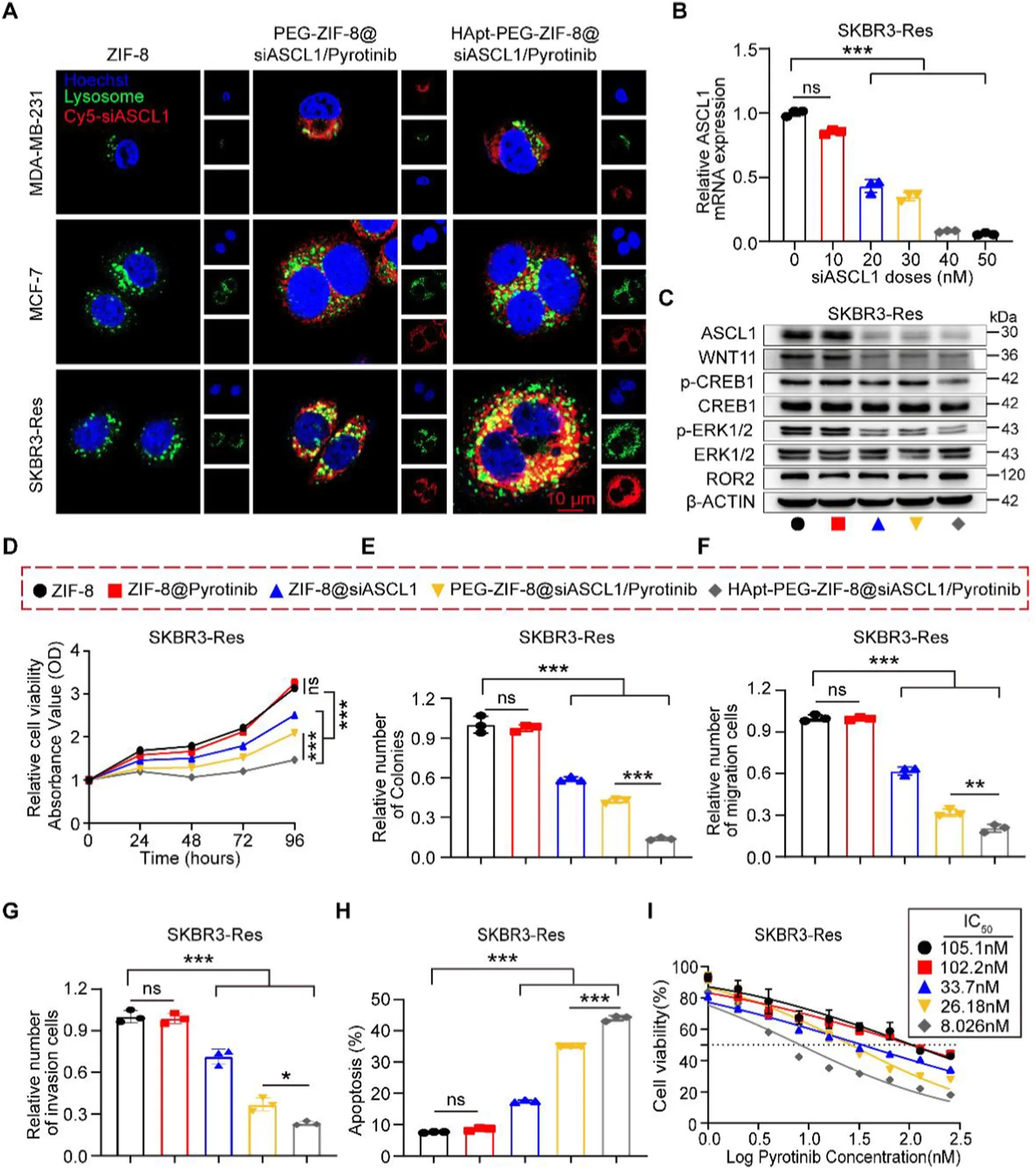
**Cellular uptake and in vitro activity of HApt-PEG-ZIF-8@siASCL1/Pyrotinib. (A)** Confocal fluorescence images showing the uptake and intracellular localization of Cy5-siASCL1 delivered by the indicated ZIF-8-based formulations in HER2-negative MDA-MB-231, HER2-low MCF-7, and HER2^+^ SKBR3-Res cells. Lysosomes were stained with LysoTracker Green, and nuclei were stained with Hoechst 33342. Scale bar, 10 μm. **(B)** RT–qPCR analysis of *ASCL1* mRNA expression in SKBR3-Res cells treated with HApt-PEG-ZIF-8@siASCL1/Pyrotinib containing increasing concentrations of siASCL1 (0–50 nM). **(C)** Immunoblot analysis of the indicated proteins in SKBR3-Res cells after treatment with the indicated formulations. **(D)** Cell viability of SKBR3-Res cells treated with the indicated formulations, measured by CCK-8 assay at the indicated time points. **(E)** Quantification of colony formation in SKBR3-Res cells following treatment with the indicated formulations. **(F, G)** Quantification of Transwell migration (F) and invasion (G) in SKBR3-Res cells following treatment with the indicated formulations. **(H)** Quantification of apoptosis in SKBR3-Res cells treated with the indicated formulations by Annexin V/PI flow cytometry. For panels C–H, siASCL1-containing formulations delivered 30 nM siASCL1, while ZIF-8@Pyrotinib and free pyrotinib added to the ZIF-8 group were dose-matched to the pyrotinib content of the dual-loaded formulations. **(I)** Pyrotinib dose–response curves in SKBR3-Res cells. All groups were matched to the indicated pyrotinib concentration; free pyrotinib was added to the ZIF-8 and ZIF-8@siASCL1 groups, with ZIF-8@siASCL1 dose-matched to the corresponding siASCL1 content of the dual-loaded formulations. IC_50_ values are indicated. Data are presented as mean ± SD. Brackets indicate the groups compared. ns, not significant; *P < 0.05, **P < 0.01, ***P < 0.001.

To assess whether HApt-mediated uptake was associated with HER2 expression, we compared the cellular uptake of Cy5-siASCL1-loaded nanoparticles in HER2-negative MDA-MB-231, HER2-low MCF-7, and HER2^+^ SKBR3-Res cells. Unloaded ZIF-8 showed only background Cy5 fluorescence. Compared with the non-targeted PEG-ZIF-8@Cy5-siASCL1/Pyrotinib formulation, HApt functionalization produced a visibly stronger intracellular Cy5 signal in SKBR3-Res cells, whereas the difference was less evident in MCF-7 and MDA-MB-231 cells. The Cy5 signal largely overlapped with LysoTracker staining, consistent with lysosomal localization after internalization (Fig. 7A). Overall, this pattern supports preferential uptake of the HApt-functionalized formulation in HER2^+^ SKBR3-Res cells. We next determined the siASCL1 concentration for subsequent experiments. HApt-PEG-ZIF-8@siASCL1/Pyrotinib containing 30 nM siASCL1 reduced *ASCL1* mRNA expression by approximately 70%. As the lowest tested concentration to achieve this degree of knockdown, 30 nM was selected for subsequent experiments (Fig. 7B).

Immunoblot analysis showed that ZIF-8 and ZIF-8@Pyrotinib had little effect on ASCL1 or WNT11 expression or on CREB1 and ERK1/2 phosphorylation. ZIF-8@siASCL1 reduced ASCL1 and WNT11 expression and decreased CREB1 and ERK1/2 phosphorylation. These effects were more pronounced with PEG-ZIF-8@siASCL1/Pyrotinib and were strongest with HApt-PEG-ZIF-8@siASCL1/Pyrotinib. Total CREB1, ERK1/2, and ROR2 levels were not appreciably altered across the different formulations (Fig. 7C). Consistent with these molecular changes, ZIF-8 and ZIF-8@Pyrotinib had little effect on cell viability, colony formation, migration, or invasion in SKBR3-Res cells. ZIF-8@siASCL1 reduced all four measures, whereas the two formulations co-delivering siASCL1 and pyrotinib produced greater inhibition. HApt-PEG-ZIF-8@siASCL1/Pyrotinib resulted in the lowest cell viability and the greatest reductions in colony formation, migration, and invasion (Fig. 7D–G). Apoptosis was also increased by ZIF-8@siASCL1 and was further increased by the two co-delivery formulations, with the highest level observed following HApt-PEG-ZIF-8@siASCL1/Pyrotinib treatment (Fig. 7H). In the pyrotinib dose–response analysis, HApt-PEG-ZIF-8@siASCL1/Pyrotinib yielded the lowest IC_50_, approximately 8.0 nM, compared with 26.2 nM for the non-targeted PEGylated formulation and 102.2 nM for ZIF-8@Pyrotinib (Fig. 7I). Together, these results indicate that co-delivery of siASCL1 and pyrotinib suppresses the ASCL1–WNT11 axis and improves pyrotinib sensitivity in SKBR3-Res cells, with further enhancement following HApt functionalization.

### 3.8 In vivo evaluation of HApt-PEG-ZIF-8@siASCL1/pyrotinib

**Fig. 8:**
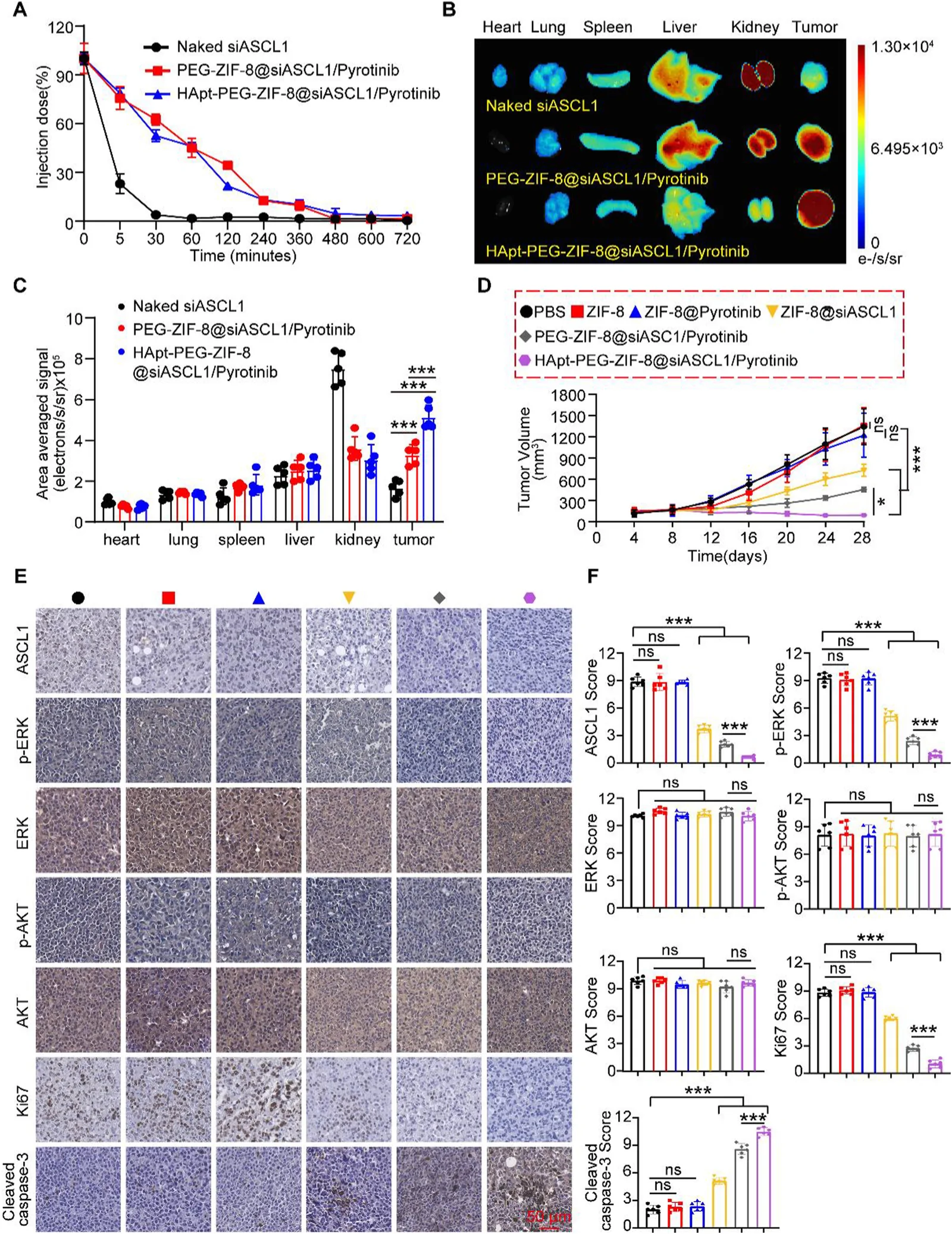
**In vivo evaluation of HApt-PEG-ZIF-8@siASCL1/Pyrotinib. (A)** Pharmacokinetic profiles of naked Cy5-siASCL1, PEG-ZIF-8@Cy5-siASCL1/Pyrotinib, and HApt-PEG-ZIF-8@Cy5-siASCL1/Pyrotinib following intravenous administration at an equivalent siASCL1 dose (n = 3 mice per group). **(B)** Representative ex vivo fluorescence images of the heart, lung, spleen, liver, kidney, and tumor collected 24 h after intravenous administration of the indicated formulations. **(C)** Quantification of Cy5 fluorescence signals in the excised organs and tumors (n = 5 mice per group). **(D)** Tumor growth curves of SKBR3-Res xenografts treated with PBS, ZIF-8, ZIF-8@Pyrotinib, ZIF-8@siASCL1, PEG-ZIF-8@siASCL1/Pyrotinib, or HApt-PEG-ZIF-8@siASCL1/Pyrotinib (n = 6 mice per group). **(E)** Representative IHC staining of ASCL1, p-ERK, ERK, p-AKT, AKT, Ki-67, and cleaved caspase-3 in excised SKBR3-Res xenografts from each treatment group. Scale bar, 50 μm. **(F)** Semiquantitative IHC scores of the indicated proteins in xenograft tumors from each group (n = 6 mice per group). Data are presented as mean ± SD. Statistical analysis was performed as described in the Methods. Brackets indicate the groups compared. ns, not significant; *P < 0.05, ***P < 0.001.

To investigate the in vivo pharmacokinetics of siASCL1, naked Cy5-siASCL1, PEG-ZIF-8@Cy5-siASCL1/Pyrotinib, and HApt-PEG-ZIF-8@Cy5-siASCL1/Pyrotinib were administered intravenously to healthy mice. Serial blood fluorescence measurements showed rapid clearance of naked Cy5-siASCL1. In contrast, both PEGylated nanoparticle formulations exhibited prolonged blood retention, with approximately 13% of the initial fluorescence signal remaining at 4 h post-injection; the two nanoparticle groups showed similar retention at this time point (Fig. 8A). To compare their in vivo distribution, the same three formulations were administered intravenously to SKBR3-Res xenograft-bearing mice. At 24 h post-injection, naked Cy5-siASCL1 showed strong renal fluorescence but relatively weak tumor fluorescence. Compared with naked Cy5-siASCL1, PEG-ZIF-8@Cy5-siASCL1/Pyrotinib showed reduced renal fluorescence, and its tumor fluorescence was approximately 1.97 times that of the naked Cy5-siASCL1 group. HApt functionalization produced a further increase in tumor accumulation, with tumor fluorescence reaching 1.56 times that of the non-targeted PEGylated formulation (Fig. 8B and C). These findings support an additional contribution of HApt functionalization to tumor accumulation.

The therapeutic efficacy of the different formulations was subsequently evaluated in SKBR3-Res xenograft models. Tumors in the PBS, ZIF-8, and ZIF-8@Pyrotinib groups grew rapidly, whereas ZIF-8@siASCL1 slowed tumor growth. PEG-ZIF-8@siASCL1/Pyrotinib produced greater tumor inhibition, and HApt-PEG-ZIF-8@siASCL1/Pyrotinib showed the strongest effect. At day 28, mean tumor volume was significantly lower in the HApt-functionalized group than in the non-targeted PEGylated group (92.6 vs 454.4 mm^3^), corresponding to a 79.6% lower mean tumor volume (Fig. 8D). Body weights increased gradually in all treatment groups, with no evident treatment-associated weight loss (Fig. S6A). IHC analysis of excised tumors was consistent with the tumor growth results. ASCL1 and p-ERK staining remained high in the PBS, ZIF-8, and ZIF-8@Pyrotinib groups, accompanied by high Ki-67 and low cleaved caspase-3 staining. ZIF-8@siASCL1 reduced ASCL1, p-ERK, and Ki-67 staining and increased cleaved caspase-3 staining. These changes were more pronounced with the two co-delivery formulations. Compared with the non-targeted PEGylated formulation, HApt-PEG-ZIF-8@siASCL1/Pyrotinib further reduced ASCL1, p-ERK, and Ki-67 staining and increased cleaved caspase-3 staining (Fig. 8E and F). These findings associate the enhanced antitumor activity of the HApt-functionalized formulation with ASCL1 suppression, reduced ERK phosphorylation and proliferation, and increased apoptosis.

Systemic toxicity was first assessed in the same antitumor efficacy cohort. Serum ALT, AST, CREA, BUN, and UA levels remained comparable across the treatment groups, and H&E staining revealed no apparent treatment-related pathological changes in the heart, liver, spleen, lung, or kidney (Fig. S6B–G). Long-term biosafety was further evaluated in a separate cohort. At 70 days after the initial administration, serum ALT, AST, BUN, and CREA levels remained comparable among mice treated with PBS, PEG-ZIF-8@siASCL1/Pyrotinib, or HApt-PEG-ZIF-8@siASCL1/Pyrotinib. H&E staining similarly revealed no apparent pathological changes in the examined major organs (Fig. S7A–E). Together, these findings indicate that both PEGylated nanoparticle formulations prolonged the blood retention of siASCL1, whereas HApt functionalization further increased tumor accumulation and antitumor activity relative to the non-targeted PEGylated formulation, without detectable alterations in the measured serum biochemical parameters or major-organ histology under the tested conditions.

## 4 Discussion

**Scheme 1:**
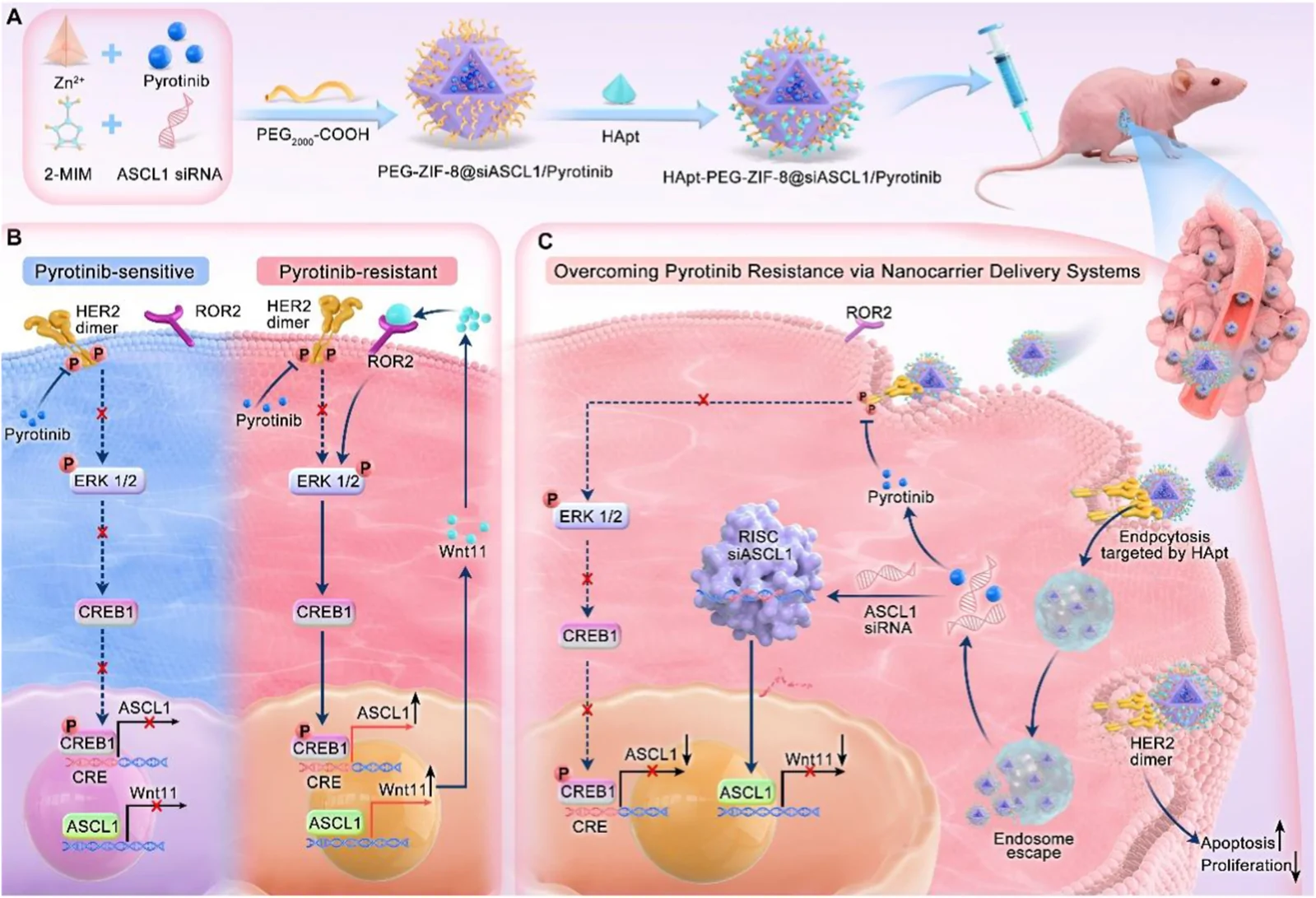
Schematic illustration of the mechanism by which HApt–PEG–ZIF-8@siASCL1/Pyrotinib nanoparticles overcome pyrotinib resistance in HER2^+^ breast cancer. In pyrotinib-resistant HER2^+^ breast cancer cells, ASCL1 is upregulated and activates WNT11–ROR2 signaling, thereby sustaining ERK activation. ERK-dependent CREB1 activation further enhances ASCL1 transcription, establishing a feed-forward resistance loop. This loop is disrupted by a HER2 aptamer–functionalized, PEGylated ZIF-8 nanocarrier that co-delivers pyrotinib and siASCL1.

Acquired resistance to pyrotinib remains a major obstacle to durable disease control in HER2^+^ BC. Here, we identify ASCL1 upregulation as a resistance-associated feature and show that ASCL1 contributes functionally to reduced pyrotinib responsiveness in vitro and in vivo. Our data support a feed-forward model in which ASCL1-driven WNT11–ROR2 signaling sustains ERK activity, while ERK-dependent CREB1 activation reinforces ASCL1 transcription, as illustrated in Scheme 1. A HER2 aptamer–functionalized ZIF-8 nanoplatform co-delivering pyrotinib and siASCL1 suppressed this program and improved treatment response in pyrotinib-resistant xenografts.

Clinically, durable responses to HER2-targeted TKIs, including pyrotinib, remain difficult to achieve, particularly in advanced and metastatic disease [35,36]. Much of the resistance literature has focused on receptor-level alterations and bypass signaling that reactivate downstream survival pathways, most notably the PI3K–AKT and MAPK arms [37]. By comparison, the contribution of lineage-associated transcriptional programs has been less well defined. ASCL1 is best known as a neuroendocrine lineage factor and oncogenic dependency in high-grade neuroendocrine lung cancers [15], and in breast pathology it has more often been discussed as part of neuroendocrine marker panels rather than as a resistance determinant [38]. Collectively, our data point to ASCL1 upregulation as a resistance-associated transcriptional state, suggesting that resistance involves more than receptor-level bypass signaling.

In an exploratory subset of 11 patients, ASCL1 expression was higher in pretreatment primary tumors from the pyrotinib-resistant group than in those from the sensitive group. In the overall cohort, high ASCL1 expression was associated with adverse clinicopathologic features and remained associated with poorer overall survival after adjustment for TNM stage. These findings suggest a possible association between pretreatment ASCL1 expression and subsequent response to pyrotinib-based therapy. However, the small, single-center pyrotinib-treated subset and lack of paired post-progression samples preclude firm predictive conclusions. Validation in a larger, independent pyrotinib-treated cohort is required to establish the predictive value of ASCL1.

Although ASCL1 is classically linked to neuroendocrine lineages, its expression has also been reported in neuroendocrine-differentiated breast carcinomas [39], indicating that this lineage factor can be engaged in breast tumors. Our functional data further suggest that ASCL1 likely contributes to the resistant phenotype, rather than merely correlating with resistance: ASCL1 overexpression reduced pyrotinib responsiveness, whereas ASCL1 knockdown improved drug response. Future studies using graded modulation of ASCL1 expression and patient-derived breast cancer models with varying endogenous ASCL1 levels will be needed to define the relationship between ASCL1 expression level and pyrotinib responsiveness.

Mechanistically, our data support a role for non-canonical WNT signaling downstream of ASCL1 in pyrotinib-resistant HER2^+^ BC. WNT11–ROR2 signaling has been implicated in invasive behavior in aggressive breast cancers and metastasis, in part through autocrine reinforcement and pro-migratory signaling [19,32]. In our models, WNT11 acted as a major ASCL1-controlled effector, and interfering with WNT11 or ROR2 reduced p-ERK and improved pyrotinib sensitivity. Notably, changes in ASCL1/WNT11 levels tracked closely with ERK phosphorylation, whereas p-AKT remained largely unchanged. This pattern is relevant because activation of MAPK signaling is a recurrent mechanism of resistance to anti-HER2 therapies in HER2^+^ BC [8], and sustained MAPK signaling can preserve proliferation and survival despite receptor inhibition. Thus, the data place WNT11–ROR2 as a route that may help maintain ERK activity during pyrotinib exposure, although its prevalence across HER2^+^ BC remains to be established.

The reciprocal question is how ASCL1 remains elevated during prolonged pyrotinib exposure. CREB-binding motifs have been reported in ASCL1 regulatory elements [33], and ERK signaling can promote CREB1 activation through Ser133 phosphorylation [34]. In our resistant models, CREB1 knockdown reduced p-CREB1 enrichment at the ASCL1 promoter and lowered ASCL1 expression, accompanied by concordant changes in WNT11 and p-ERK. Mutation of the predicted CREB1-binding motif prevented CREB1-induced activation of the ASCL1 promoter, providing direct functional evidence that this promoter element is required for CREB1-dependent transcription. In parallel, MEK–ERK inhibition reduced CREB1 phosphorylation, ASCL1 promoter activity, and endogenous *ASCL1* mRNA expression without altering *ASCL1* mRNA decay. Together, these observations support a predominantly transcriptional mechanism through which persistent ERK activity maintains ASCL1 expression via CREB1. However, two quantitative relationships in the proposed resistance mechanism remain unresolved, one between p-CREB1 occupancy at the ASCL1 promoter and endogenous ASCL1 transcriptional output, and the other, as previously discussed in the context of the OE-ASCL1 model, between ASCL1 expression and pyrotinib responsiveness. Notably, the ASCL1–CREB1 relationship may not be fixed across treatment contexts. A recent report linked ASCL1 to paclitaxel response via a ferroptosis-related CREB1/GPX4 program [17], suggesting that the relative positioning of ASCL1 and CREB1 may shift under different therapeutic pressures. More directly, transcriptional reprogramming has been observed as HER2^+^ BC cells transition from drug-tolerant to proliferative states under continuous HER2 inhibition, and DUSP6–HER3 signaling can support HER2-inhibitor tolerance [40]. These findings are consistent with the broader view that durable HER2 TKI resistance can involve downstream adaptive programs in addition to receptor-level alterations.

From a translational perspective, the key challenge is to suppress ASCL1-associated resistance while maintaining HER2 blockade, ideally in a tumor-selective manner. Although transcription factors are difficult to target directly with small molecules, other components of this axis may be more amenable to therapeutic intervention. ROR2 has also emerged as a druggable surface target in breast cancer, including antibody-based and antibody–drug conjugate strategies [[41], [42], [43]]. In parallel, we pursued a delivery strategy to enable ASCL1 silencing alongside pyrotinib exposure. ZIF-8 has been widely investigated for nucleic-acid delivery and pH-responsive release behavior, which can support intracellular payload liberation in acidic compartments [44,45], and ZIF-8-based designs have also been used for co-delivery paradigms combining gene-silencing payloads with anti-cancer interventions [46]. Recent ZIF-8 systems have also used pH-responsive co-encapsulation of complementary bioactive components, including glucose oxidase and DNA-templated silver nanoclusters, to generate cascade antitumor effects [47]. We incorporated PEGylation to reduce nonspecific interactions and improve in vivo performance [26,48], and used a HER2-targeting aptamer to enhance selectivity for HER2-expressing cells [27], consistent with broader progress in HER2-directed aptamer/siRNA delivery concepts [49].

Representative HER2-targeted nanocarriers include trastuzumab-functionalized ZIF-8 carrying doxorubicin [50], an HB5-functionalized PEGylated mesoporous silica–carbon platform combining doxorubicin delivery with NIR-triggered photothermal therapy [51], trastuzumab-functionalized mesoporous silica nanoparticles co-delivering docetaxel and siHER2 [52], and trastuzumab-functionalized AuAg hollow nanoshells combining pyrotinib delivery with NIR-triggered photothermal therapy [53]^.^ These systems establish precedents for the individual carrier, targeting, co-delivery, and therapeutic components relevant to our platform. Its distinguishing feature is the mechanism-guided integration: HB5-mediated HER2 recognition with pH-responsive co-delivery of pyrotinib and siASCL1 to address the ASCL1-associated mechanism of pyrotinib resistance identified here. Unlike trastuzumab, which can contribute both HER2 recognition and therapeutic HER2 inhibition, HB5 serves as the targeting ligand, whereas pyrotinib and siASCL1 provide the therapeutic activities. The present formulation also does not require external irradiation.

Within the matched formulations evaluated in this study, the tumor-associated Cy5-siASCL1 fluorescence signal at 24 h with HApt functionalization was 1.56-fold that of the PEGylated formulation without HApt (*P* < 0.001), and the mean tumor volume at day 28 was 79.6% lower (*P* < 0.05). Both formulations retained approximately 13% of the initial blood fluorescence signal at 4 h. These findings quantify the incremental effect of HApt functionalization within this formulation pair. However, because no head-to-head comparison with an antibody-functionalized carrier was performed and the published systems differ in carrier composition, payloads, dosing schedules, external activation, and tumor models, superiority over antibody-based strategies or other published platforms cannot be inferred.

Although the in vitro release profiles demonstrate pH-dependent pyrotinib release from the nanoplatform, the chemical stability of pyrotinib under the tested pH conditions was not directly assessed. The reported EE and LC values were obtained from a single nanoparticle batch. Future studies will prepare multiple independent batches and determine EE and LC for each batch. Both tumor-bearing model systems were established by subcutaneous implantation of human SKBR3-derived cells in female BALB/c nude mice. Although these models provided in vivo evidence for ASCL1-associated pyrotinib resistance and the antitumor activity of the co-delivery formulation, they do not provide an intact adaptive immune context and do not reproduce the mammary-site microenvironment or metastatic disease [54]. Vascular characteristics relevant to nanocarrier delivery can also differ between ectopic and orthotopic breast tumor xenografts [55]. Consequently, the present study cannot determine how immune and site-specific microenvironmental factors influence pyrotinib resistance, nanocarrier delivery, or therapeutic response. Future studies should use orthotopic and metastatic models to assess site-specific microenvironmental effects and appropriately selected immunocompetent or humanized models to examine immune contributions.

## 5 Conclusion

In summary, this study identifies ASCL1 as a functional mediator of pyrotinib resistance and reveals a preliminary association between pretreatment ASCL1 expression and clinical response to pyrotinib-based therapy in HER2^+^ breast cancer. Our data suggest that ERK-dependent CREB1 activity is linked to ASCL1 transcription and to an ASCL1–WNT11–ROR2 signaling module associated with persistent ERK phosphorylation during pyrotinib exposure. Building on this mechanism, co-delivery of ASCL1 siRNA and pyrotinib using a HER2-directed, ZIF-8–based nanocarrier markedly improved therapeutic response in pyrotinib-resistant xenograft models, with minimal short-term systemic toxicity and no detectable alterations in the measured serum biochemical parameters or major-organ histology at the 70-day endpoint.

## CRediT authorship contribution statement

**Fang Yin:** Writing – original draft, Methodology, Investigation, Visualization. **Boxuan Zhou:** Writing – original draft, Methodology. **Yingliang Li:** Data curation. **Zide Liu:** Data curation, Formal analysis. **Chao Zhu:** Methodology. **Yu Wang:** Methodology. **Chuang Meng:** Methodology. **Zhanglin Zhang:** Methodology. **Alan Jiang:** Methodology. **Wei Liu:** Methodology. **Hongfei Liu:** Methodology. **Xiaocheng Mao:** Validation. **Hong Tang:** Methodology. **Tianmei Fu:** Methodology. **Linwei Fan:** Methodology. **Chengqi Gao:** Methodology, Investigation. **Kuai Yu:** Methodology. **Qi Zeng:** Writing – original draft, Data curation, Writing – review & editing. **Aiping Le:** Writing – review & editing, Writing – original draft.

## Declaration of competing interest

The authors declare that they have no known competing financial interests or personal relationships that could have appeared to influence the work reported in this paper.

## Data Availability

Data will be made available on request.
