# Supplementary material for: Targeting an ASCL1–WNT11–ERK feed-forward loop to overcome pyrotinib resistance in HER2-positive breast cancer
Source: Mater Today Bio. 2026 Sep 17;41:103679. doi: 10.1016/j.mtbio.2026.103679 (PMC13602770; doi:10.1016/j.mtbio.2026.103679)
Supplement: Supplementary file 1 [file mmc1.docx]

Supplementary Materials for

Targeting an ASCL1–WNT11–ERK Feed-Forward Loop to Overcome Pyrotinib Resistance in HER2-positive Breast Cancer

Fang Yin^1,#^, Boxuan Zhou^1,2,3,#^, Yingliang Li^2^, Zide Liu^4^, Chao Zhu^1^, Yu Wang^1^, Chuang Meng^1,3^, Zhanglin Zhang^1^, Alan Jiang^5^, Wei Liu^1^, Hongfei Liu^1^, Xiaocheng Mao^1,6^, Hong Tang^1^, Tianmei Fu^1^, Linwei Fan^7^, Chengqi Gao^1,3,^*, Kuai Yu^1,^*, Qi Zeng^1,^*, Aiping Le^1,^*

Correspondence to: [ndyfy00973@ncu.edu.cn](mailto:ndyfy00973@ncu.edu.cn)

**This supplementary file includes:**

Figures. S1 to S7

Tables. S1 to S6

**Figure S1. High ASCL1 expression is associated with poor prognosis in breast cancer. (A)** Representative phase-contrast images showing morphological changes in SKBR3-Sen and SKBR3-Res cells. Scale bar, 20 μm. **(B)** Dose–response curves of SKBR3-Sen and SKBR3-Res cells treated with pyrotinib, with IC_50_ values indicated. **(C)** Kaplan–Meier overall survival analyses of ASCL1, IL17REL, PIP, RGPD1, and KCNH2 in an independent HER2^+^ BC cohort using the KM-Plotter online database; hazard ratios (HRs) and log-rank *P* values are shown.

**Figure S2. ASCL1 knockdown restores pyrotinib sensitivity in SKBR3-Res cells. (A)** RT–qPCR analysis of *ASCL1* mRNA expression in SKBR3-Res cells transduced with sh-NC or shASCL1-1 and shASCL1-2. **(B)** Immunoblot analysis of indicated proteins in SKBR3-Res cells expressing sh-NC, shASCL1-1, or shASCL1-2. **(C)** Dose–response curves of SKBR3-Res cells treated with pyrotinib following ASCL1 knockdown; IC_50_ values are indicated. **(D)** Cell viability of SKBR3-Res sh-NC, shASCL1-1, and shASCL1-2 cells treated with DMSO or pyrotinib, measured at the indicated time points. **(E)** Representative colony formation images and quantification of SKBR3-Res sh-NC, shASCL1-1, and shASCL1-2 cells under DMSO or pyrotinib treatment. **(F)** EdU incorporation assay showing representative fluorescence images and quantification of EdU-positive cells in SKBR3-Res sh-NC, shASCL1-1, and shASCL1-2 cells treated with DMSO or pyrotinib. Scale bar, 100 μm. **(G)** Transwell migration and invasion assays showing representative images and quantification of SKBR3-Res sh-NC, shASCL1-1, and shASCL1-2 cells treated with DMSO or pyrotinib. Scale bar, 100 μm. **(H)** Flow cytometric analysis of apoptosis by Annexin V/PI staining in SKBR3-Res sh-NC, shASCL1-1, and shASCL1-2 cells treated with DMSO or pyrotinib, with quantification of apoptotic cells. Data are presented as mean ± SD. Statistical analysis was performed as described in the Methods. Brackets indicate the groups compared. ns, not significant. **P* < 0.05, ***P* < 0.01, ****P* < 0.001.

**Figure S3. Validation of the ASCL1–WNT11–ROR2 signaling axis. (A)** Spearman correlation analysis between ASCL1 and WNT11 IHC scores in clinical HER2^+^ breast cancer tissues (n = 36). **(B)** RT–qPCR validation of WNT11 knockdown efficiency in SKBR3-Sen cells transduced with WNT11-targeting shRNAs. **(C)** Cell viability of SKBR3-Res shASCL1 cell line, with or without WNT11 overexpression (rescue), under pyrotinib treatment. **(D)** Quantification of transwell migration in SKBR3-Res shASCL1 cell line, with or without WNT11 rescue, under pyrotinib treatment. **(E)** Quantification of apoptosis (Annexin V/PI staining) in SKBR3-Res shASCL1 cell line, with or without WNT11 rescue, under pyrotinib treatment. **(F)** Dose–response curves to pyrotinib in SKBR3-Res shASCL1 cell line, with or without WNT11 rescue; IC_50_ values were calculated. **(G)** RT–qPCR validation of *ROR2* knockdown efficiency in SKBR3-Res cells transduced with ROR2-targeting shRNAs. **(H)** Dose–response curves to pyrotinib in SKBR3-Res cells following ROR2 knockdown; IC_50_ values were calculated. Data are presented as mean ± SD. Statistical analysis was performed as described in the Methods. Brackets indicate the groups compared. ns, not significant; **P* < 0.05, ***P* < 0.01, ****P* < 0.001.

**Figure S4. CREB1 regulates WNT11 expression and pyrotinib resistance through ASCL1. (A)** RT–qPCR validation of CREB1 knockdown in SKBR3-Res cells transduced with sh-NC, shCREB1-1, shCREB1-2, or shCREB1-3. **(B)** RT–qPCR analysis of the indicated mRNA levels in SKBR3-Sen cells with ASCL1 overexpression. **(C, D)** SKBR3-Res cells transduced with sh-NC or shCREB1, with or without ASCL1 re-expression, were analyzed under pyrotinib treatment. **(C)** Cell viability measured by CCK-8 assay; the indicated group comparisons were performed at 96 h. **(D)** Quantification of transwell migration. **(E)** ChIP–qPCR analysis of p-CREB1 enrichment at the WNT11 promoter in SKBR3-Res cells transduced with sh-NC, shCREB1-1, or shCREB1-2. IgG served as a negative control. **(F)** Luciferase reporter assay in HEK-293T cells showing WNT11 promoter activity following CREB1 overexpression, with or without ASCL1 knockdown. Data are presented as mean ± SD. Statistical analyses were performed as described in the Methods. Brackets indicate the groups compared. ns, not significant; ****P* < 0.001.

**Figure S5. UV–Vis analysis of pyrotinib and Cy5-labeled siASCL1 loading and HApt conjugation. (A, B)** UV–Vis absorption spectra of pyrotinib standard solutions (1–10 μg/mL) and the supernatant collected after pyrotinib loading (A), together with the corresponding calibration curve constructed from the absorbance at 260.5 nm (B). **(C, D)** UV–Vis absorption spectra of Cy5-labeled siASCL1 standard solutions (5–80 μg/mL) and the supernatant collected after Cy5-labeled siASCL1 loading (C), together with the corresponding calibration curve constructed from the absorbance at 646.5 nm (D). **(E, F)** UV–Vis absorption spectra of HApt standard solutions (1–10 μg/mL) and the supernatant collected after HApt conjugation to the PEGylated nanoparticles (E), together with the corresponding calibration curve constructed from the absorbance at 261 nm (F). The three supernatants were collected and analyzed separately after the corresponding loading or conjugation steps. Black dashed curves denote the respective supernatants, and gray dotted vertical lines indicate the wavelengths used for quantification.

**Figure S6. Systemic toxicity assessment in the antitumor efficacy cohort (A)** Body weight of SKBR3-Res xenograft-bearing mice in different treatment groups during therapy (n = 6, mean ± SD). **(B–F)** Blood biochemical tests, including alanine aminotransferase (ALT) (B), aspartate aminotransferase (AST) (C), creatinine (CREA) (D), blood urea nitrogen (BUN) (E), and uric acid (UA) (F), after different treatments (n = 6, mean ± SD). **(G)** H&E staining of the heart, liver, spleen, lung, and kidney after different treatments. Scale bar: 100 μm.

**Figure S7. Long-term biosafety evaluation. (A–D)** Serum biochemical parameters, including alanine aminotransferase (ALT) (A), aspartate aminotransferase (AST) (B), blood urea nitrogen (BUN) (C), and creatinine (CREA) (D), measured 70 days after the first administration in mice treated with PBS, PEG-ZIF-8@siASCL1/Pyrotinib, or HApt-PEG-ZIF-8@siASCL1/Pyrotinib (n = 6, mean ± SD). **(E)** Representative H&E staining images of the heart, liver, spleen, lung, and kidney collected 70 days after the first administration. Scale bar: 100 μm.

**Table S1.** Clinicopathological characteristics of 146 patients with HER2-positive breast cancer.

| Characteristics | Total cases (n = 146) | ASCL1 expression level | | |
| --- | --- | --- | --- | --- |
|  |  | Low (n = 73) | High (n = 73) | *P* value |
| **Age, years** |  |  |  |  |
| ≤ 50 | 76 | 39 | 37 | 0.740 |
| > 50 | 70 | 34 | 36 |  |
| **Menopausal status** |  |  |  |  |
| Pre | 64 | 34 | 30 | 0.505 |
| Post | 82 | 39 | 43 |  |
| **Pathological grade** |  |  |  |  |
| ≤ G2 | 85 | 41 | 44 | 0.615 |
| > G2 | 61 | 32 | 29 |  |
| **TNM stage** |  |  |  |  |
| I–II | 114 | 64 | 50 | 0.005** |
| III–IV | 32 | 9 | 23 |  |
| **T stage** |  |  |  |  |
| T1–2 | 128 | 67 | 61 | 0.131 |
| T3–4 | 18 | 6 | 12 |  |
| **lymph node status** |  |  |  |  |
| Negative | 86 | 51 | 35 | 0.007** |
| Positive | 60 | 22 | 38 |  |
| **ER** |  |  |  |  |
| Negative | 73 | 37 | 36 | 0.869 |
| Positive | 73 | 36 | 37 |  |
| **PR** |  |  |  |  |
| Negative | 94 | 44 | 50 | 0.300 |
| Positive | 52 | 29 | 23 |  |
| **Ki-67** |  |  |  |  |
| Low | 22 | 8 | 14 | 0.165 |
| High | 124 | 65 | 59 |  |

*P* values were calculated using χ² or Fisher’s exact tests, as appropriate. ***P* < 0.01.

**Table S2.** Univariate and multivariate Cox regression analyses of overall survival in 146 patients with HER2-positive breast cancer (24 deaths).

|  | Univariate analysis ^a^ |  | Multivariate analysis ^b^ | |  |
| --- | --- | --- | --- | --- | --- |
| Variables | HR (95% CI) | *P* value | HR (95% CI) | *P* value | |
| **Age** (> 50 vs. ≤ 50) | 1.639 (0.730–3.682) | 0.232 | — | — | |
| **Menopausal status**  ( post vs. pre) | 2.323 (0.961–5.616) | 0.061 | — | — | |
| **Pathological grade**  (> G2 vs. ≤ G2) | 0.900 (0.394–2.056) | 0.802 | — | — | |
| **TNM stage**  (III–IV vs. I–II) | 5.292 (2.230–12.559) | < 0.001*** | 4.347 (1.806–10.464) | 0.001** | |
| **T stage**  ( T3–4 vs. T1–2) | 3.250 (1.284–8.225) | 0.013* | — | — | |
| **Lymph node status** (Positive vs. Negative ) | 4.520 (1.912–10.683) | 0.001** | — | — | |
| **ER status**  (ER^+^ vs. ER^-^) | 0.701 (0.311–1.579) | 0.391 | — | — | |
| **PR status**  (PR^+^ vs. PR^-^) | 0.596 (0.236–1.502) | 0.272 | — | — | |
| **Ki-67 positive rate**  (High vs. Low) | 1.943 (0.456–8.280) | 0.369 | — | — | |
| **ASCL1 score**  (High vs. Low) | 4.084 (1.524–10.942) | 0.005** | 3.380 (1.248–9.155) | 0.017* | |

a. Cox proportional hazards regression was used.

b. The multivariate model included ASCL1 expression and TNM stage. T stage and lymph node status were not entered separately because both are components of TNM stage. An em dash indicates a variable not included in the multivariate model. HR, hazard ratio; CI, confidence interval.

**P* < 0.05; ***P* < 0.01; ****P* < 0.001.

| **Formulation** | **Zeta potential (mV)** | **Hydrodynamic diameter (nm)** | **Polydispersity index (PDI)** |
| --- | --- | --- | --- |
| ZIF-8 | 29.50 ± 0.76 | 116.9 ± 10.6 | 0.280 ± 0.068 |
| ZIF-8@Pyrotinib | 23.88 ± 1.19 | 250.3 ± 8.0 | 0.277 ± 0.059 |
| ZIF-8@siASCL1 | −28.98 ± 0.50 | 175.0 ± 2.5 | 0.044 ± 0.017 |
| PEG-ZIF-8@siASCL1/Pyrotinib | −26.93 ± 0.77 | 257.2 ± 6.4 | 0.279 ± 0.011 |
| HApt-PEG-ZIF-8  @siASCL1/Pyrotinib | −23.53 ± 0.57 | 344.9 ± 17.0 | 0.168 ± 0.108 |

**Table S3.** Physicochemical characteristics of the ZIF-8-based nanoparticle formulations.

Data are presented as mean ± SD from three replicate measurements.

**Table S4.** Primer sequences for RT-qPCR, siRNA, shRNA, promoter cloning and ChIP-qPCR.

1. **RT-qPCR**

| β-actin | F | 5′-CACCATTGGCAATGAGCGGTTC-3′ |  |
| --- | --- | --- | --- |
| ASCL1  IL17REL  PIP | R  F  R  F  R  F  R | 5′-AGGTCTTTGCGGATGTCCACGT-3′  5′-CGGCCAACAAGAAGATGAGT-3′  5′-GCCATGGAGTTCAAGTCGTT-3′  5′-CCCCTTTGAAAACGACACTGA-3′  5′-GTACTGCACTCTGCGATGC-3′  5′-GTCAGTACGTCCAAATGACGAA-3′  5′-CTGTTGGTGTAAAAGTCCCAGT-3′ |  |
| RGPD1 | F | 5′-ACCACCTCGATGGGCAGAAGAT-3′ |  |
| KCNH2  WNT11  ROR2 | R  F  R  F  R  F  R | 5′-TATCCATCAGGCACCGAGTCTG-3′  5′-CAACCTGGGCGACCAGATAG-3′  5′-GGTGTTGGGAGAGACGTTGC-3′  5′-GACCTCAAGACCCGATACCTG-3′  5′-TAGACGAGTTCCGAGTCCTTC-3′  5′-GCACAGACTTCCCTGAGCTT-3′  5′-TGCTGTCTCGGGGACTACAC-3′ |  |
| CREB1 | F | 5′-AGCTTGTACCACCGGTAACT-3′ |  |
|  | R | 5′-ACCTGGGCTAATGTGGCAAT-3′ |  |

2. **siRNA**

| siNC  siASCL1 | Sense  Anti-sense  Sense  Anti-sense | 5’-UUCUCCGAACGUGUCACGUTT-3′  5’-ACGUGACACGUUCGGAGAATT-3′  5’-CCAACAAGAAGAUGAGUAATT-3′  5’-UUACUCAUCUUCUUGUUGGTT-3′ |
| --- | --- | --- |

3. **shRNA**

| shASCL1-1 | Sense | | 5’- CCAACAAGAAGATGAGTAATTCAAGAGATTACTCATCTTCTTGTTGGTTTTTTGAATTC-3′ |
| --- | --- | --- | --- |
|  | Anti-sense | 5’-GAATTCAAAAAACCAACAAGAAGATGAGTAATCTCTTGAATTACTCATCTTCTTGTTGG-3′ | |
| shASCL1-2 | Sense | 5’- ACTACTCCAACGACTTGAATTCAAGAGATTCAAGTCGTTGGAGTAGTTTTTTTGAATTC-3′ | |
|  | Anti-sense | 5’- GAATTCAAAAAAACTACTCCAACGACTTGAATCTCTTGAATTCAAGTCGTTGGAGTAGT-3′ | |
| shWNT11-1 | Sense | 5’-GATCGGCACTGAACCAGACGCAACACTCGAGTGTTGCGTCTGGTTCAGTGCCTTTTTG-3′ | |
|  | Anti-sense | 5’-AATTCAAAAAGGCACTGAACCAGACGCAACACTCGAGTGTTGCGTCTGGTTCAGTGCC-3′ | |
| shWNT11-2 | Sense | 5’-GATCGCGACAGCTGCGACCTTATGTCTCGAGACATAAGGTCGCAGCTGTCGCTTTTTG-3′ | |
|  | Anti-sense | 5’-AATTCAAAAAGCGACAGCTGCGACCTTATGTCTCGAGACATAAGGTCGCAGCTGTCGC-3′ | |
| shROR2-1 | Sense | 5’-GATCGGTCCAACGCACAGCCCAAATCTCGAGATTTGGGCTGTGCGTTGGACCTTTTTG-3′ | |
|  | Anti-sense | 5’-AATTCAAAAAGGTCCAACGCACAGCCCAAATCTCGAGATTTGGGCTGTGCGTTGGACC-3′ | |
| shROR2-2 | Sense | 5’-GATCGCTACCATCAGTGCTATAACGCTCGAGCGTTATAGCACTGATGGTAGCTTTTTG-3′ | |
|  | Anti-sense | 5’-AATTCAAAAAGCTACCATCAGTGCTATAACGCTCGAGCGTTATAGCACTGATGGTAGC-3′ | |
| shROR2-3 | Sense | 5’-GATCGCCTCTTCCGAGAGGTGTATGCTCGAGCATACACCTCTCGGAAGAGGCTTTTTG-3′ | |
|  | Anti-sense | 5’-AATTCAAAAAGCCTCTTCCGAGAGGTGTATGCTCGAGCATACACCTCTCGGAAGAGGC-3′ | |
| shCREB1-1 | Sense | 5’-GATCGCAGCTCATGCAACATCATCTCTCGAGAGATGATGTTGCATGAGCTGCTTTTTG-3′ | |
|  | Anti-sense | 5’-AATTCAAAAAGCAGCTCATGCAACATCATCTCTCGAGAGATGATGTTGCATGAGCTGC-3′ | |
| shCREB1-2 | Sense | 5’-GATCGCGGCCCAGCCATCAGTTATTCTCGAGAATAACTGATGGCTGGGCCGCTTTTTG-3′ | |
|  | Anti-sense | 5’-AATTCAAAAAGCGGCCCAGCCATCAGTTATTCTCGAGAATAACTGATGGCTGGGCCGC-3′ | |
| shCREB1-3 | Sense  Anti-sense | 5’-GATCGCCAACTCCAATTTACCAAACCTCGAGGTTTGGTAAATTGGAGTTGGCTTTTTG-3′  5’-AATTCAAAAAGCCAACTCCAATTTACCAAACCTCGAGGTTTGGTAAATTGGAGTTGGC-3′ | |

4. **Dual-luciferase reporter assay (promoter cloning)**

| WNT11-promoter | F | 5’- GTCACTATCCTTCCCCCATGTG-3′ |
| --- | --- | --- |
|  | R | 5’- CTCCTCCTCGCCTGGCG-3′ |
| ASCL1- promoter | F | 5’- GGTGCCCAGGCTGTGTTG-3′ |
|  | R | 5’-TGAGAGAGTGCTGGGCTGC-3′ |

5. **ChIP-qPCR**

| ASCL1 IP, WNT11 promoter | F  R | 5’-CACCTCATTAACTCCCTTCCCA-3′  5’-CCTTTTCCAACTCCCCAAACC-3′ |
| --- | --- | --- |
| p-CREB1 IP, ASCL1 promoter | F  R | 5’- TCGCCGGTCTCATCCTACTC-3′  5’- CAAAGTCCATTCGCACCAGG-3′ |
| p-CREB1 IP, WNT11 promoter | F | 5’- GGCTGGGGTCAAGTCCTCTT-3′  5’- GTCCTATAGAACGACCAGGACAC-3′ |
|  | R |  |

**Table S5.** List of primary antibodies used for immunoblotting.

| Antibodies | Dilution ratio | Source | Cat No. |
| --- | --- | --- | --- |
| ASCL1 | 1:1000 | CST | 43666 |
| WNT11 | 1:1000 | Huabio | ER1918-68 |
| Phospho-ERK1/2 (Thr202/Tyr204) | 1:2000 | Proteintech | 28733-1-AP |
| ERK1/2 | 1:2000 | Proteintech | 11257-1-AP |
| Phospho-AKT (Ser473) | 1:2000 | Proteintech | 66444-1-Ig |
| AKT | 1:5000 | Proteintech | 60203-2-Ig |
| Phospho-CREB (Ser133) | 1:1000 | CST | 9198 |
| CREB1 | 1:2000 | Proteintech | 12208-1-AP |
| ROR2 | 1:1000 | Huabio | HA721417 |
| β-ACTIN | 1:10000 | Proteintech | 66009-1-Ig |
| GAPDH | 1:20000 | Huabio | ET1601-4 |
| HA | 1:10000 | ABclonal | AE008 |
| DYKDDDDK | 1:5000 | Proteintech | 66008-4-Ig |

**Table S6.** List of antibodies used for immunohistochemistry (IHC) staining.

| Antibody | Dilution ratio | Source | Cat No. |
| --- | --- | --- | --- |
| ASCL1 | 1:100 | Abcam | ab211327 |
| WNT11 | 1:300 | Huabio | ER1918-68 |
| Phospho-ERK1/2 (Thr202/Tyr204) | 1:200 | Proteintech | 28733-1-AP |
| ERK1/2 | 1:200 | Proteintech | 11257-1-AP |
| Phospho-AKT (Ser473) | 1:200 | Proteintech | 66444-1-Ig |
| AKT | 1:300 | Proteintech | 60203-2-Ig |
| Ki-67 | 1:5000 | Huabio | HA721115 |
| Cleaved caspase-3 | 1:300 | Proteintech | 25128-1-AP |
